# Macrophage membrane-reversibly camouflaged nanotherapeutics accelerate fracture healing by fostering MSCs recruitment and osteogenic differentiation

**DOI:** 10.1186/s12951-024-02679-y

**Published:** 2024-07-12

**Authors:** Cheng Wu, Jing Yan, Chenglong Ge, Lucheng Xie, Yunjie He, Ziyin Zhao, Yekun Deng, Qirong Dong, Lichen Yin

**Affiliations:** 1 Department of Orthopedics, The Second Affiliated Hospital, Soochow University, Suzhou, 215004 China; 2 Department of Orthopedics, The Affiliated Hospital of Guizhou Medical University, Guiyang, 550004 China; 3 Department of Gastroenterology, The Second Affiliated Hospital, Soochow University, Suzhou, 215004 China; 4 Institute of Functional Nano and Soft Materials (FUNSOM), Jiangsu Key Laboratory of Carbon-Based Functional Materials and Devices, Soochow University, Suzhou, 215123 China

**Keywords:** Bone fracture repair, siRNA delivery, Endogenous MSCs recruitment, Osteogenesis, Cell membrane-cloaked nanotherapeutics

## Abstract

The fracture healing outcome is largely dependent on the quantities as well as osteogenic differentiation capacities of mesenchymal stem cells (MSCs) at the lesion site. Herein, macrophage membrane (MM)-reversibly cloaked nanocomplexes (NCs) are engineered for the lesion-targeted and hierarchical co-delivery of short stromal derived factor-1α peptide (sSDF-1α) and Ckip-1 small interfering RNA (Ckip-1 siRNA, siCkip-1) to promote bone repair by concurrently fostering recruitment and osteogenic differentiation of endogenous MSCs. To construct the NCs, a membrane-penetrating α-helical polypeptide first assembles with siCkip-1, and the cationic NCs are sequentially coated with catalase and an outer shell of sSDF-1α-anchored MM. Due to MM-assisted inflammation homing, intravenously injected NCs could efficiently accumulate at the fractured femur, where catalase decomposes the local hydrogen peroxide to generate oxygen bubbles that drives the shedding of sSDF-1α-anchored MM in the extracellular compartment. The exposed, cationic inner core thus enables robust trans-membrane delivery into MSCs to induce Ckip-1 silencing. Consequently, sSDF-1α-guided MSCs recruitment cooperates with siCkip-1-mediated osteogenic differentiation to facilitate bone formation and accelerate bone fracture healing. This study provides an enlightened strategy for the hierarchical co-delivery of macromolecular drugs into different cellular compartments, and it also renders a promising modality for the management of fracture healing.

**Supplementary Information:**

The online version contains supplementary material available at 10.1186/s12951-024-02679-y.

## Introduction

Bone fracture is a public health issue around the world, and millions of bone fractures occur annually [1, 2]. Delayed fracture healing will lead to nonunion and long-term disabilities [3, 4]. Under current therapeutic regimens, approximately 10% of patients suffer from delayed fracture healing or nonunion [5, 6]. Therefore, medical interventions that can accelerate the fracture repair is of great significance, which will contribute to the reduction of the incident rate of fracture-related complications (such as nonunion) [4, 7–9]. Mesenchymal stem cells (MSCs), the progenitor cells of osteoblasts, have shown tremendous potentials in facilitating bone repair and regeneration [10–14]. In particular, MSCs, together with other cells (bone progenitor cells, stromal cells, and macrophages), participate in the cascaded events in bone repair and regeneration, including inflammation, repair, and remodeling [15–18]. Moreover, MSCs can differentiate into osteoblasts and then secrete major components of extracellular bone matrix such as osteocalcin (OCN), osteopontin (OPN), alkaline phosphatase (ALP), and type I collagen (Col I) to promote the in situ deposition of calcium ions, thus facilitating the mineralization of extracellular matrix and finally leading to the formation of hard tissues [19–21]. Therefore, maintaining adequate quantities of MSCs with robust osteoblast differentiation capabilities in the lesion site is of critical importance for bone repair [22].

Supplement of exogenous MSCs and recruitment of endogenous MSCs are two major approaches for increasing the quantities of MSCs in the diseased site [23–26]. The former approach, nevertheless, is often challenged by their limited source, stringent yet complex in vitro culture condition, infection-associated side effect, immunological rejection, low in vivo cell survival, and tumorigenic risk [27–30]. In comparison, recruitment of endogenous MSCs using chemokines such as stromal derived factor-1α (SDF-1α), C-C motif chemokine ligand 25, and C-X-C motif chemokine ligand 16, represents a safer and more efficient modality for fracture repair [31–35]. sSDF-1α (KSKPVVLSYR), a homologue to the receptor activating domain of SDF-1α, retains the full capability of SDF-1α in recruiting MSCs [36–38], and hence targeted immobilization of sSDF-1α to the fracture site can guide the homing of MSCs and foster fracture healing.

Effective osteogenic differentiation of MSCs is another critical issue for fracture healing [39–41]. Disordered or hindered osteogenic differentiation reduces the production of extracellular matrix proteins, impedes matrix mineralization, and retards bone remodeling [42, 43]. After bone fracture, the interruption of local blood supply leads to the generation of lactic acid and a pro-inflammatory condition, thus creating an acidic and reactive oxygen species (ROS)-enriched microenvironment [44–47]. Such an environment results in the dysfunction of MSCs, inhibiting their intrinsic osteogenic differentiation capabilities *via* generation of the osteogenic inhibitory molecules and intervention of signaling pathways associated with osteogenesis [48, 49]. Casein kinase-2 interacting protein-1 (Ckip-1) is a recently identified negative regulator of osteogenesis in MSCs [50, 51]. It enhances the ligase activity of Smurf1 for proteasomal degradation of Smad 1/5, the critical signaling molecules in the bone morphogenetic protein (BMP) signaling pathway, which makes it a potential molecular target for the management of bone fracture [52, 53]. As thus, down-regulation of Ckip-1 *via* small interfering RNA (siRNA)-mediated gene silencing renders a promising strategy to induce osteogenic differentiation of MSCs, which can be further coupled with sSDF-1-mediated MSCs recruitment to accelerate fracture healing.

Efficient and fracture-targeted co-delivery of sSDF-1α and Ckip-1 siRNA (siCkip-1) is critical for realizing their cooperative functions. However, sSDF-1α and siCkip-1 function extracellularly and intracellularly, respectively, which makes their co-delivery challenging. Positively charged polymers are often utilized to aid trans-membrane siRNA delivery, but they suffer from instability during blood circulation and are prone to be cleared by the reticuloendothelial system [54–57]. Surface-coating with cell membranes has been developed as an effective tool to neutralize the positive surface charges of nanovehicles and accordingly enhances the serum stability. Moreover, certain cell membranes can endow the nanovehicles with distinct biological functions. For instance, immune cell (such as macrophage or neutrophil) membrane can promote the homing to the inflamed tissues (such as the fractured bones); red blood cell membrane can favor long circulation in the blood [58–60]. However, membrane-decorated nanovehicles often suffer from greatly compromised trans-membrane delivery capabilities [59, 61]. Therefore, a membrane-shedding mechanism at the pathological site is highly desired to enable efficient cytosolic delivery of siRNA cargoes.

**Scheme 1:**
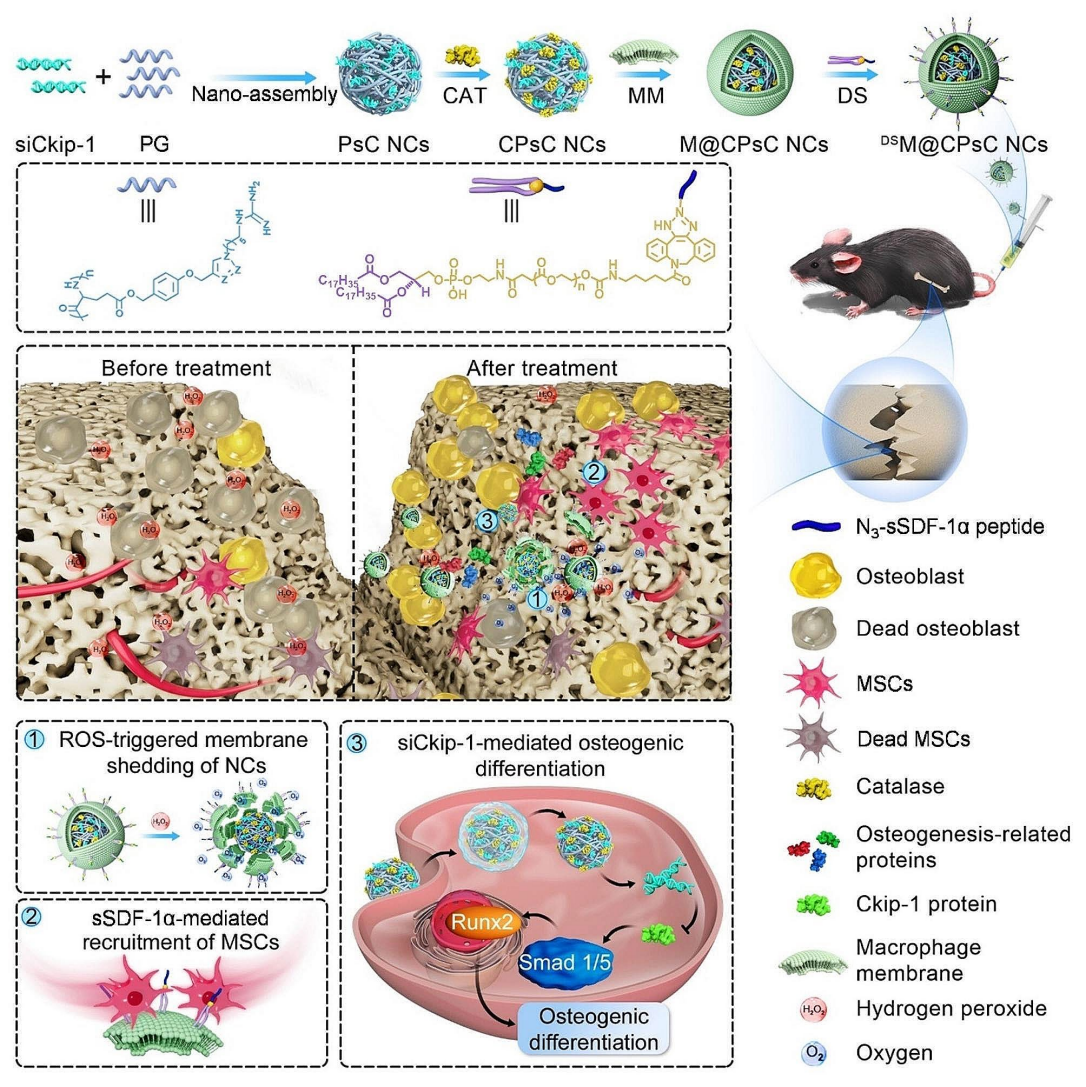
Schematic illustration showing the fabrication and hierarchical co-delivery of siCkip-1 and sSDF-1α for promoting fracture healing. Cationic helical polypeptide (PG) complexed with siCkip-1 to form the positively charged inner core, which adsorbed negatively charged CAT and was further coated with DS-anchored MM to form the final ^DS^M@CPsC NCs. After *i.v.* injection to femur-fractured mice, the NCs could effectively accumulate in the lesion site due to MM-assisted inflammation homing. Then, CAT inside the NCs decomposed the over-produced H_2_O_2_ in the inflamed microenvironment to generate oxygen bubbles and shed off the DS-anchored MM, thus exposing the positively charged inner core to facilitate intracellular delivery and Ckip-1 silencing in MSCs. As such, sSDF-1α-assisted MSCs recruitment cooperated with siCkip-1-mediated osteogenic differentiation to promote bone formation and facilitate fracture healing

Based on the above understandings, nanocomplexes (NCs) reversibly cloaked with macrophage membrane (MM) were herein developed to mediate the targeted co-delivery of sSDF-1α and siCkip-1 and hierarchical cargo release in the fractured site. The NCs consisted of a cationic inner core constructed from the membrane-penetrating, α-helical polypeptide (PG) and siCkip-1, an intercalated catalase (CAT) middle layer, and an outer shell based on MM anchored with 1,2-dioleoyl-sn-glycero-3-phosphoethanolamine (DSPE)-modified sSDF-1α. Upon systemic administration into femur-fractured mice, the NCs could accumulate in the inflamed fracture site based on the MM-assisted inflammation homing. In the inflammatory microenvironment, CAT catalyzed the decomposition of over-produced hydrogen peroxide (H_2_O_2_), generating large quantities of oxygen bubbles to drive the shedding of the outer membrane shell, such that sSDF-1α located in the extracellular compartment could recruit MSCs to the fracture site. Meanwhile, the exposed cationic NCs efficiently transfected MSCs and silenced Ckip-1 expression, hence activating the Smad 1/5-Runx2 pathway to induce osteogenic differentiation. The increased MSCs infiltration and enhanced osteogenic differentiation cooperated to accelerate the bone fracture healing (Scheme 1).

## Methods

### Materials

CAT was purchased from Beyotime Biotechnology (Shanghai, China). 1,2-Dioleoyl-sn-glycero-3-phosphoethanolamine-conjugated diphenyl-poly(ethylene glycol)_2k_ monomethyl ether-cyclooctyne (DSPE-PEG_2k_-DBCO) was purchased from Ruixi Biological Technology Co., LTD. (Xi’an, China). α-Helical polypeptide bearing guanidine groups on the side chains (PG, polymerization degree = 102) was synthesized according to our previous report [54]. Lysotracker Red and Trizol reagent were purchased from Thermo Fisher Scientific (Massachusetts, USA). BCA protein assay kit, 3-(4,5-dimethyl-2-thiazolyl)-2,5-diphenyl tetrazolium bromide (MTT), and 4*’*,6-diamidino-2-phenylindole (DAPI) were purchased from Alfa Aesar (Shanghai, China). PrimerScript real-time reagent kit and SYBR Premix Ex Taq kit were purchased from Takara Bio (Qingdao, China). Azide-modified sSDF-1α peptide (N_3_-KSKPVVLSYR, N_3_-sSDF-1α) and Cy3-labeled N_3_-sSDF-1α were purchased from Bankpeptide Biotechnology Co., LTD. (Hefei, China). Primary antibodies (anti-Col I, anti-ALP, anti-Runx2, anti-OCN, and anti-OPG) were purchased from Solarbio Life Sciences Co., LTD. (Beijing, China) or Beyotime Biotechnology (Shanghai, China). Secondary antibodies of horseradish peroxidase-conjugated goat anti-mouse IgG and goat anti-rabbit IgG were purchased from Absin Bioscience Co., LTD. (Shanghai, China). APC-labeled anti-CD105, PE-labeled anti-CD73, and Percy/Cy5.5-labeled anti-CD45 were purchased from Elabscience Biotechnology Co., LTD (Wuhan, China). siCkip-1 and negative control siRNA with scrambled sequence (siScr) were purchased from GenePharma Co., LTD. (Shanghai, China), and their sequences were listed in Supplementary: Table S1. All primers were purchased from Sangong Biotech (Shanghai, China), and their sequences were listed in Supplementary: Table S2. All the other reagents were purchased from Energy Chemical (Shanghai, China) or Aladdin (Shanghai, China) and used as received.

### Animals and cells

Female C57BL/6 mice (6–8 weeks, 18–20 g) were purchased from Slaccas Experimental Animal Co., LTD. (Shanghai, China) and housed in a specific pathogen-free (SPF) animal lab.

RAW 264.7 cells (mouse monocyte macrophage) were purchased from the American Type Culture Collection (Rockville, MD, USA) and cultured in DMEM containing 10% fetal bovine serum (FBS). Mouse MSCs (bone marrow mesenchymal stem cells) were purchased from Cyagen Biosciences Co., LTD. (Guangzhou, China) and cultured in α-MEM containing 10% FBS.

### Mouse femur fracture model

The femur fracture model was established according to a previous report [43]. Particularly, mice were anesthetized, and their right hips, thighs, and knees were sterilized with povidone-iodine solution. A 2-cm medial parapatellar incision was created and the patella was dislocated to expose the femoral condyles. A hole was drilled into the femoral intramedullary canal at the intercondylar notch using a 25-gauge needle to stabilize the impending fracture. Immediately after the needle implantation, blunt dissection of muscle was performed to expose the midshaft of the femur, and a transverse femoral shaft fracture was then created in the right femur of each mouse using a rotary Dremel with a diamond blade attachment. The patella was then reduced, and the incision was closed using 4 − 0 synthetic suture. Mice were allowed to move freely after recovery from anesthesia. On day 5 post fracture, animals were imaged with an X-ray imaging system (Faxitron MX-20, Tucson, AZ) to verify that the mid-diaphyseal fracture in femur had been produced.

### Cell membrane isolation

RAW 264.7 cell membrane as the MM was isolated according to a previous report [62]. Briefly, RAW 264.7 cells on the culture dish (60 mm in diameter) were harvested and suspended in the homogenization buffer containing Tris·HCl (pH 7.5, 20 mM), potassium chloride (10 mM), sucrose (75 mM), magnesium chloride (2 mM), and protease/phosphatase inhibitors. The suspension was disrupted with a probe ultrasonic disruptor (JY 92-IIN, Ningbo Scientz, 100 W, sonicate for 5 s and pause for 5 s, 10 min, 4 ºC) and then centrifuged (20,000 g, 25 min). The supernatant was centrifuged (100,000 g, 35 min) again, and the pellet was collected as the RAW 264.7 cell membrane and stored at -80 ºC until use. The protein content of MM was determined using the BCA protein assay kit. For fluorescence microscopy imaging and fluorescence resonance energy transfer (FRET) analysis, DiO-stained MM and DiI-stained MM were prepared by mixing the cell membrane with DiO or DiI at the membrane protein/dye weight ratio of 1000/1 [59].

### Preparation and characterization of NCs

PG was prepared according to our previous report [54]. The chemical structure and the secondary structure of PG were determined by ^1^H NMR and CD, respectively. Then, PG solution (1 mg/mL in DEPC water) was mixed with siCkip-1 solution (0.1 mg/mL in DEPC water) at various PG/siCkip-1 weight ratios (2.5, 5, 10, 15, 20, and 25). The mixture was vortexed for 5 s and incubated at room temperature (RT) for 20 min to form PG/siCkip-1 (PsC) NCs. Then, CAT solution (4 mg/mL) was added to PsC NCs at various CAT/siCkip-1 weight ratios (2.5, 5, 10, and 15), vortexed for 5 s, and incubated for 20 min to obtain the CAT-adsorbed PsC NCs (CPsC NCs). Subsequently, MM-coated CPsC NCs (M@CPsC NCs) were fabricated using the sonication method as reported previously [58]. MM solution (5 mg/mL) was added to CPsC NCs at various membrane protein/siCkip-1 weight ratios (5, 10, 15, and 20), followed by sonication (2 min) to allow membrane coating. The freshly prepared NCs were subjected to electrophoresis (90 V, 20 min) in agarose gel (2%) to observe the siRNA migration. The zeta potential and hydrodynamic diameter of the freshly prepared NCs were recorded on a Zetasizer (Nano ZS 90, Malvern). The morphology of NCs was observed by transmission electron microscopy (TEM) following negative staining with phosphotungstic acid (1%, w/v). The stability of NCs in PBS (pH 7.4) containing 10% FBS was evaluated by measuring the particle size following incubation at RT for various time. To determine the membrane coating efficiency, DiD-stained MM was coated onto CPsC NCs as described above. Then, the obtained ^DiD^M@CPsC NCs were centrifuged (10,000 g, 10 min), and the amount of un-coated DiD-stained MM in the supernatant was determined by spectrofluorimetry (*λ*_ex_ = 644 nm, *λ*_em_ = 665 nm). The fluorescence intensity of the freshly prepared ^DiD^M@CPsC NCs before centrifugation was determined and set as 100%.

To prepare sSDF-1α-immobilized M@CPsC NCs (^DS^M@CPsC NCs), DSPE-PEG_2k_-conjugated sSDF-1α (DS) was firstly prepared *via* the click reaction between DSPE-PEG_2k_-DBCO and N_3_-sSDF-1α. Briefly, DSPE-PEG_2k_-DBCO solution (10 mg/mL in PBS, pH = 7.4, 224 µL) was mixed with N_3_-sSDF-1α solution (5 mg/mL in PBS, pH = 7.4, 200 µL) and stirred at 37 °C for 2 h. DS was obtained after purification by ultrafiltration (MWCO = 3 kDa). The purified solution was collected and subjected to high performance liquid chromatography (HPLC, Thermofisher) analysis equipped with a UV-vis detector (*λ*_abs_ = 214 nm) to determine the sSDF-1α concentration in the final DS solution. A mixture of acetonitrile and water (4:1, v/v) containing 0.1% trifluoroacetic acid was used as the mobile phase. The freshly prepared DS was mixed with M@CPsC NCs at various membrane protein/sSDF-1α weight ratios, vortexed for 5 s, and incubated for 10 min to obtain ^DS^M@CPsC NCs. The bovine serum albumin (BSA)-containing NCs (^DS^M@BPsC NCs) were similarly prepared, wherein BSA was used instead of CAT. The abbreviations of various NCs were listed in Table S3. FRET assay was conducted to confirm the insertion of DS into the cell membrane. Particularly, ^DS^M@CPsC NCs were constructed from DiO-labeled MM and Cy3-labeled DS at various membrane protein/sSDF-1α weight ratios. As a control, M@CPsC NCs (containing DiO-labeled MM) were mixed with Cy3-labeled N_3_-sSDF-1α (without DSPE as the membrane-anchoring domain) instead of Cy3-labeled DS. The fluorescence emission spectrum of each sample was recorded between 520 and 600 nm at the excitation wavelength of 480 nm. Macrophage-specific surface markers (MAC-1 and CD68) on MM and ^DS^M@CPsC NCs were examined by Western blot.

### In vitro oxygen generation and gas-driven membrane shedding

Free CAT, ^DS^M@CPsC NCs, and ^DS^M@BPsC NCs (0.1 mg CAT or BSA/mL) were incubated with H_2_O_2_ (50 mM) at 37 °C for 1 h. The generation of oxygen bubbles was recorded by a digital camera.

To monitor the membrane shedding, freshly prepared ^DS^M@CPsC NCs and ^DS^M@BPsC NCs were treated with H_2_O_2_ (100 µM) for different time, followed by measurement of the size and zeta potential. Then, the FRET assay was also conducted. Briefly, ^DS^M@CPsC NCs and ^DS^M@BPsC NCs comprised of DiI-labeled MM and FAM-siCkip-1 were incubated with H_2_O_2_ (100 µM) for 4 h. The fluorescence emission spectra of NCs before and after H_2_O_2_ treatment were recorded between 500 and 650 nm at the excitation wavelength of 494 nm. The fluorescence recovery of the donor (FAM) at 530 nm was used to represent the membrane shedding from NCs. Finally, confocal laser scanning microscopy (CLSM, Zeiss LSM 800) was used to observe the membrane shedding. The freshly prepared ^DS^M@CPsC NCs comprised of FAM-siCkip-1 and DiI-labeled MM were treated with H_2_O_2_ (100 µM) for 4 h followed by CLSM observation.

### In vitro MSCs migration

The transwell culture system was adopted to evaluate the sSDF-1α-mediated MSCs migration. Briefly, MSCs were seeded onto the apical side of the inserts (pore size of 8.0 μm, Corning, NY, 1 × 10^6^ cell/mL) and cultured for 24 h. Then, the cell culture medium at the basolateral side was replaced with fresh α-MEM containing sSDF-1α, H_2_O_2_-treated (100 μm, 4 h) ^DS^M@CPsS NCs, or untreated ^DS^M@CPsS NCs (1 µg siScr/mL, 1 µg sSDF-1α/mL). After incubation for 48 h, the culture medium at both the apical and basolateral sides was replaced with neutral formalin (10%) and incubated for 10 min. Then, cells at the basolateral side of the transwell membrane were stained with crystal violet (0.1%, 30 min), washed with PBS for three times, and observed by an optical microscope. Six fields at 20× magnification were randomly selected to count the number of migrated MSCs.

### Cellular uptake and intracellular distribution of NCs in MSC

MSCs were seeded on 6-well plates (3 × 10^5^ cells/well) and cultured for 24 h. Then, various FAM-siCkip-1-containing NCs, including CPsC NCs, ^DS^M@CPsC NCs, H_2_O_2_-treated (100 µM, 4 h) ^DS^M@CPsC NCs, and H_2_O_2_-treated (100 µM, 4 h) ^DS^M@BPsS^FAM^ NCs, were added at the final concentration of 1 µg FAM-siCkip-1/mL. After 4-h incubation, cells were washed with PBS for three times, re-suspended in PBS (0.3 mL), and subjected to flow cytometric (FCM, FACS Calibur, BD, USA) analysis. Data were analyzed using the Flowjo software.

The endo/lysosomal escape of NCs was observed by CLSM. MSCs were seeded on glass-bottomed dishes (2 × 10^4^ cells/dish, 20 mm in diameter) and cultured for 24 h. Cells were then incubated with H_2_O_2_-treated (100 µM, 4 h), FAM-siCkip-1-containing ^DS^M@CPsC NCs at 1 µg FAM-siCkip-1/mL for 4 h. After washing with PBS containing sodium heparin (20 U /mL) for three times, cells were stained with Lysotracker Red (200 nM, 1.5 h) and Hoechst 33342 (10 µg/mL, 20 min) followed by CLSM observation. The co-localization ratios were analyzed using the Image J software.

### In vitro cytotoxicity of NCs

MSCs were seeded on 96-well plates (8 × 10^3^ cells/well) and cultured for 24 h. Various NCs were added at 1 µg siCkip-1/mL and incubated with cells for 24 h. The cell viability was then determined by the MTT assay. Cells treated with PBS were used as the control to represent 100% viability.

### In vitro Ckip-1 silencing and osteogenesis

MSCs were seeded on 6-well plates (1 × 10^5^ cells/well) and cultured for 24 h. After replacement with fresh medium, ^DS^M@CPsC NCs, H_2_O_2_-treated (100 µM, 4 h) ^DS^M@CPsS NCs, or H_2_O_2_-treated (100 µM, 4 h) ^DS^M@CPsC NCs were added at 1 µg siRNA/mL and incubated with cells for 24 h. The Ckip-1 mRNA level in cells was determined by real-time PCR. To evaluate the osteogenic differentiation of MSCs, the mRNA levels of Smad 1/5 and osteogenesis-associated genes (Runx2, Col I, ALP, and OCN) were determined by real-time PCR. Moreover, Ckip-1 and osteogenesis-associated proteins (Runx2, Col I, ALP, and OCN) levels were also determined by Western blot. The concentrations of primary antibody and second antibody were both 1/1000. GAPDH was used as the internal control.

To further explore the NCs-mediated osteogenic differentiation of MSCs, MSCs were seeded on 6-well plates (1 × 10^5^ cells/well) and cultured for 24 h. After replacement with osteo-induction medium, H_2_O_2_-treated (100 µM, 4 h) ^DS^M@CPsC NCs or ^DS^M@CPsS NCs were added at 1 µg siRNA/mL. The medium containing various NCs was refreshed every 2 d. After 14-d incubation, cells were washed with PBS for three times, fixed with neutral formalin (10%, 10 min), and stained by Alizarin red S (ARS, 5 mg/mL, 15 min) to show calcium deposition. Cells were washed with PBS for three times and imaged using an inverted microscope (Leica TSR2). Cells were then incubated with cetylpyridinium chloride (10%, pH = 7.0) for 15 min, and subjected to determination of absorbance at 562 nm using a microplate reader (Bio-Tek, Synergy H1).

### Pharmacokinetics, biodistribution, and fracture-targeting of NCs in vivo

C57BL/6 mice were *i.v.* injected with Cy5-siCkip-1-containing CPsC NCs or ^DS^M@CPsC NCs (1 mg Cy5-siCkip-1/kg, 1 mg sSDF-1α/kg). At predetermined time points, blood (70 µL) was collected from the orbit and mixed with the passive lysis buffer (100 µL, supplemented with 1% Triton X-100). Dimethyl sulfoxide (DMSO, 200 µL) was added into the mixture and incubated overnight at RT. After centrifugation (14,800 rpm, 30 min), the concentration of Cy5-siCkip-1 in the supernatant was determined by spectrofluorimetry (*λ*_ex_ = 633 nm, *λ*_em_ = 678 nm).

For the evaluation of the in vivo targeting of fractured femur, femur-fractured C57BL/6 mice were *i.v.* injected with Cy5-siCkip-1-containing ^DS^M@CPsC NCs or CPsC NCs (1 mg Cy5-siCkip-1/kg, 1 mg sSDF-1α/kg) at 24 h post fracture. At predetermined time intervals (1, 3, 6, 9, 12, and 24 h), mice were imaged using the Maestro In Vivo Imaging System. In a parallel study, mice were sacrificed at 24 h post injection. The major organs (heart, liver, spleen, lung, and kidney) and the whole femurs were harvested and imaged (*λ*_ex_ = 633 nm, *λ*_em_ = 678 nm).

### In vivo photoacoustic (PA) imaging

Femur-fractured C57BL/6 mice were *i.v.* injected with PBS, ^DS^M@BPsC NCs, or ^DS^M@CPsC NCs (1 mg siCkip-1/kg, 1 mg sSDF-1α/kg) at 24 h post fracture. The echo signal from O_2_ in the fractured femur was recorded using the PA imaging system (FujiFilm VisualSonics Inc.) with the PA mode (Oxy-hem mode, 750 and 850 nm) at 3 h post injection.

### In vivo membrane shedding

The in vivo membrane shedding from NCs was monitored by the FRET assay. Femur-fractured C57BL/6 mice were *i.v.* injected with ^DS^M@CPsC NCs or ^DS^M@BPsC NCs comprised of Cy5-siCkip-1 and DiI-labeled MM (1 mg Cy5-siCkip-1/kg). At 6 h post injection, mice were sacrificed, and the fractured femurs were harvested and imaged using the Maestro In Vivo Imaging System (*λ*_ex_ = 550 nm, *λ*_em_ = 670 nm). The disappearance of the acceptor (Cy5) signal was used to represent the separation of MM and siCkip-1.

### In vivo uptake of NCs by MSCs

Femur-fractured C57BL/6 mice were *i.v.* injected with FAM-siCkip-1-containing NCs (^DS^M@CPsC NCs and ^DS^M@BPsC NCs) at 1 mg FAM-siCkip-1/kg. At 24 h post injection, mice were sacrificed, and the fractured femurs were harvested, flushed with sterile PBS, grinded with a mortar, and digested with enzymes (2 mg/mL collagenase, 100 µg/mL DNase, and 20 µg/mL RNase) for 1 h to prepare mono-dispersed cell suspensions. Cells were collected *via* centrifugation (500 g, 3 min), stained with antibodies (PerCP/Cy5.5-labeled anti-CD45, PE-labeled anti-CD73, and APC-labeled anti-CD105) for 0.5 h, washed with PBS for three times, resuspended in the FACS buffer (PBS containing 1% FBS, 0.2 mL), and subjected to FCM analysis. Cells were first gated using FSC/SSC, followed by CD45, CD73, and CD105 gating to identify CD45^−^CD73^+^CD105^+^ populations (MSCs) for the determination of MSCs that had taken up FAM-siCkip-1-containing NCs.

### In vivo MSCs recruitment, Ckip-1 silencing, and osteogenesis

Femur-fractured C57BL/6 mice were *i.v.* injected with PBS, ^DS^M@CPsS NCs, ^DS^M@CPsC NCs, or M@CPsC NCs (1 mg siRNA/kg, 1 mg sSDF-1α/kg) on day 1, 3, and 5 post fracture. Five millimeter-length bones spanning the fracture sites were harvested on day 7, flushed with sterile PBS, grinded with a mortar, and digested with enzymes (2 mg/mL collagenase, 100 µg/mL DNase, and 20 µg/mL RNase) for 1 h to prepare mono-dispersed cell suspensions. Cells were then washed with PBS, stained with antibodies (PerCP/Cy5.5-labeled anti-CD45, PE-labeled anti-CD73, and APC-labeled anti-CD105), washed with PBS, and subjected to FCM analysis. In a parallel study, femurs were harvested on day 7 or 28 post fracture. The mRNA levels of Ckip-1 and osteogenesis-associated genes (Runx2, Col I, ALP, and OCN) in femurs on day 7 were determined by real-time PCR following reported protocols [59]. The Ckip-1 protein level on day 7 was further determined by Western blot as described before [54]. The expression levels of osteogenesis-associated proteins (Runx2, Col I, ALP, OCN, and OPG) on day 28 were determined by immunofluorescence (IFC) or immunohistochemical (IHC) staining and quantified using the ImageJ software. Data were calculated as the mean fluorescence intensity (for IFC) or mean gray value (for IHC) of the positive cells.

### X-ray and micro-CT imaging

Femur-fractured C57BL/6 mice were *i.v.* injected with ^DS^M@CPsS NCs, ^DS^M@CPsC NCs, or M@CPsC NCs (1 mg siRNA/kg, 1 mg sSDF-1α/kg) on day 1, 3, and 5 post fracture. Mice were imaged with an X-ray imaging system (Faxitron MX-20, Tucson, AZ) on day 14 and 28. The radiographs on day 28 were scored for callus opacity, cortical remodeling/bridging, and periosteal/endosteal reaction by three independent assessors blinded to grouping and total scores were calculated [63]. Callus opacity and periosteal/endosteal reaction were scored by the apparent radiographic density and the significance level of periosteal/endosteal reaction, respectively. Scoring range for both callus opacity and periosteal/endosteal reaction went from 0 (none) to 3 (marked). Cortical remodeling/bridging was scored by visibility and mineralization of cortical edges, and scores ranged from 0 (none) to 4 (complete union with well demarcated medullary canal).

In a parallel study, femurs were harvested on day 28 post fracture and scanned using micro-CT (Skyscan 1176, Belgium) as described previously [62]. High resolution scanograms (9–20 mm) were obtained (resolution: 8.8 mm, source voltage: 50 kV, source current: 500 mA, rotation step: 0.7 unit). The data set was reconstructed using the CT analyzer software (Skyscan) to obtain the 3D images of femur and to measure morphometric parameters. The region of interest (ROI) in the fractured femur was chosen for the determination of bone mineral density (BMD), trabecular number (Tb.N), and trabecular separation/spacing (Tb.Sp).

### Histological analysis

Femurs were harvested on day 28 post fracture as described above. Excess soft tissues and skin were removed. Femurs were fixed in 4% neutral formalin for 3 d, decalcified in 10% ethylenediaminetetraacetic acid solution for one month at RT, embedded in paraffin, and sliced at 6 μm in thickness. The femur sections were stained with Sirius red, Masson’s trichrome (MT), haematoxylin and eosin (H&E), or Safranin O/fast green to evaluate the Col I expression, total collagen deposition, new bone formation, and bone mineralization, respectively, as important indexes for osteogenesis. The contents of Col I (bright red/yellow collagen fiber), deposited total collagen (dark blue), newly formed bone (including lamellar bone and cartilage), and mineralized bone (green area) were determined using the ImageJ software. Data were denoted as the percentage of stain-positive area to the total area in each section.

### Biosafety assessment of ^DS^M@CPsC NCs

Healthy C57BL/6 mice were *i.v.* injected with PBS or ^DS^M@CPsC NCs (1 mg siCkip-1/kg) for three times with 24 h spaced between each injection. Blood was collected at 24 h post the last injection followed by hematological and biochemical analyses. Major organs were also harvested and subjected to histological assessment using H&E staining.

### Statistical analysis

All data were presented as means ± standard deviations, and statistical analysis was performed using One-way ANOVA. The differences between two experimental groups were judged to be significant at *p < 0.05 and very significant at **p < 0.01 and ***p < 0.001.

## Results and discussion

### Preparation and characterization of NCs

**Fig. 1:**
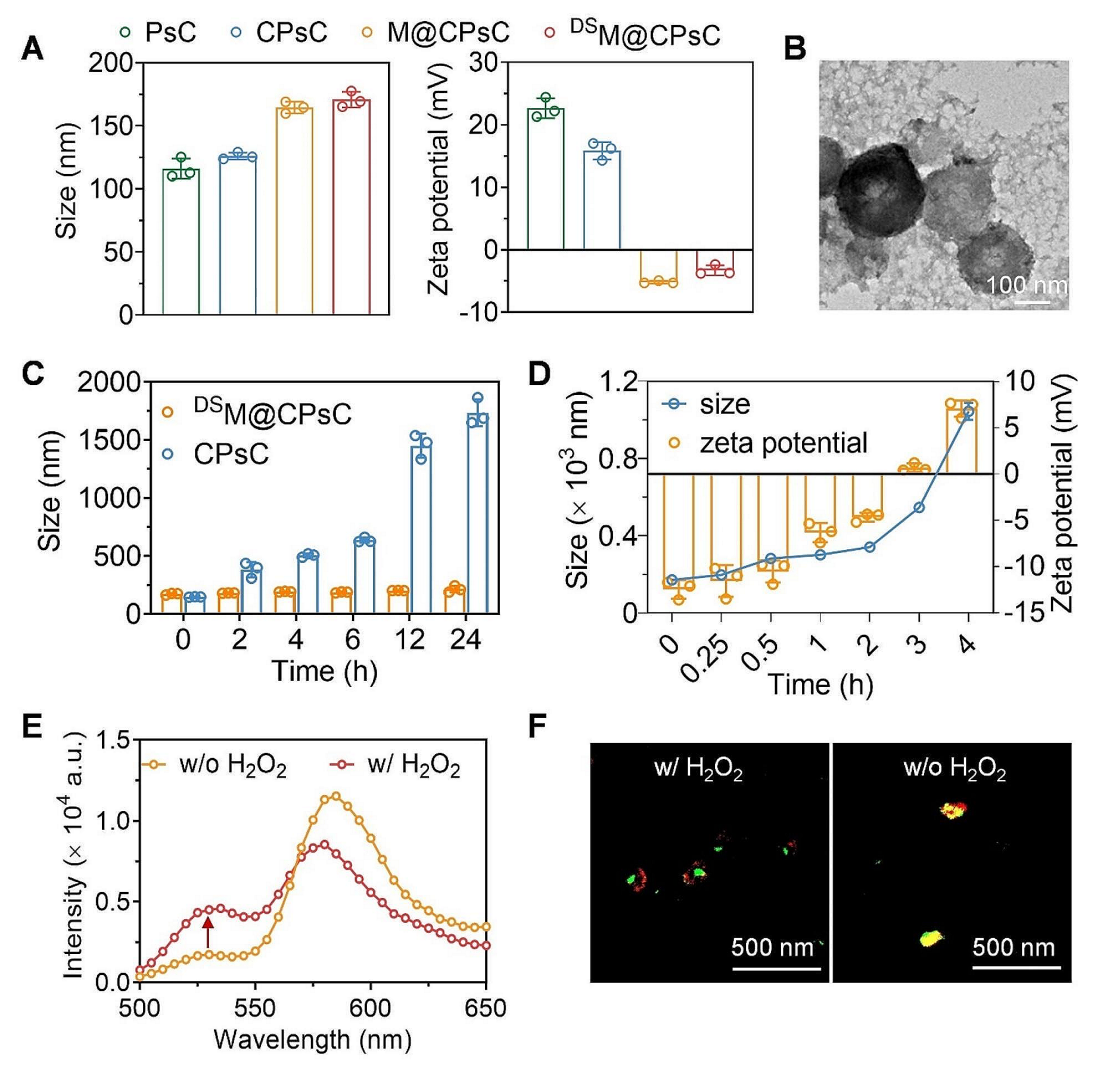
Characterization of NCs and H_2_O_2_-mediated membrane shedding. (**A**) Size and zeta potential of various NCs (w/w/w/w, PG/CAT/MM/siCkip-1 = 10/2.5/10/1, *n* = 3). (**B**) TEM image of ^DS^M@CPsC NCs. (**C**) Size of CPsC NCs and ^DS^M@CPsC NCs after incubation in PBS containing 10% FBS for various time (*n* = 3). (**D**) Size and zeta potential of ^DS^M@CPsC NCs after treatment with H_2_O_2_ (100 µM) for various time (*n* = 3). Fluorescence emission spectra (*λ*_ex_ = 494 nm, **E**) and CLSM images (**F**) of ^DS^M@CPsC NCs comprised of FAM-siCkip-1 and DiI-MM with (w/) or without (w/o) H_2_O_2_ treatment (100 µM, 4 h)

PG was prepared according to a previous report [54]. ^1^H NMR spectrum confirmed the chemical structure of PG (Supplementary: Figure S1). The CD spectrum of PG in the aqueous solution revealed double minima at 208 and 222 nm, indicating the α-helical conformation (Supplementary: Figure S2) [64]. Then, PG allowed to complex with siCkip-1 *via* electrostatic interaction and salt bridging to form the binary PG/siCkip-1 NCs (PsC NCs). siRNA migration after agarose gel electrophoresis was retarded at the PG/siCkip-1 weight ratios ≥ 5 (Supplementary: Figure S3A). Increase of the PG/siCkip-1 weight ratio led to decrease of the particle size yet increase of the zeta potential, and at the weight ratio of 10, PsC NCs with the diameter of 116 nm and zeta potential of + 22.6 mV were obtained (Supplementary: Figure S3B, 3 C, and Fig. 1A). Considering the potential toxicity of the cationic polypeptide at excessive amount, PG/siCkip-1 weight ratio of 10 was adopted to prepare PsC NCs. At such PG/siCkip-1 weight ratio, PsC NCs adsorbed the negatively charged CAT *via* electrostatic interaction to obtain the ternary CAT/PG/siCkip-1 NCs (CPsC NCs). At incremental CAT/siCkip-1 weight ratios from 0 to 10, no siCkip-1 migration was observed after electrophoresis, indicating that siCkip-1 was not repelled out of the NCs after CAT adsorption (Supplementary: Figure S4A). The particle size of the ternary CPsC NCs kept increasing along with the increase of the CAT/siCkip-1 weight ratio, and NCs with hydrodynamic diameter of 126 nm and positive zeta potential of + 15.9 mV were obtained at the weight ratio of 2.5 (Supplementary: Figure S4B, 4 C, and Fig. 1A).

**Fig. 2:**
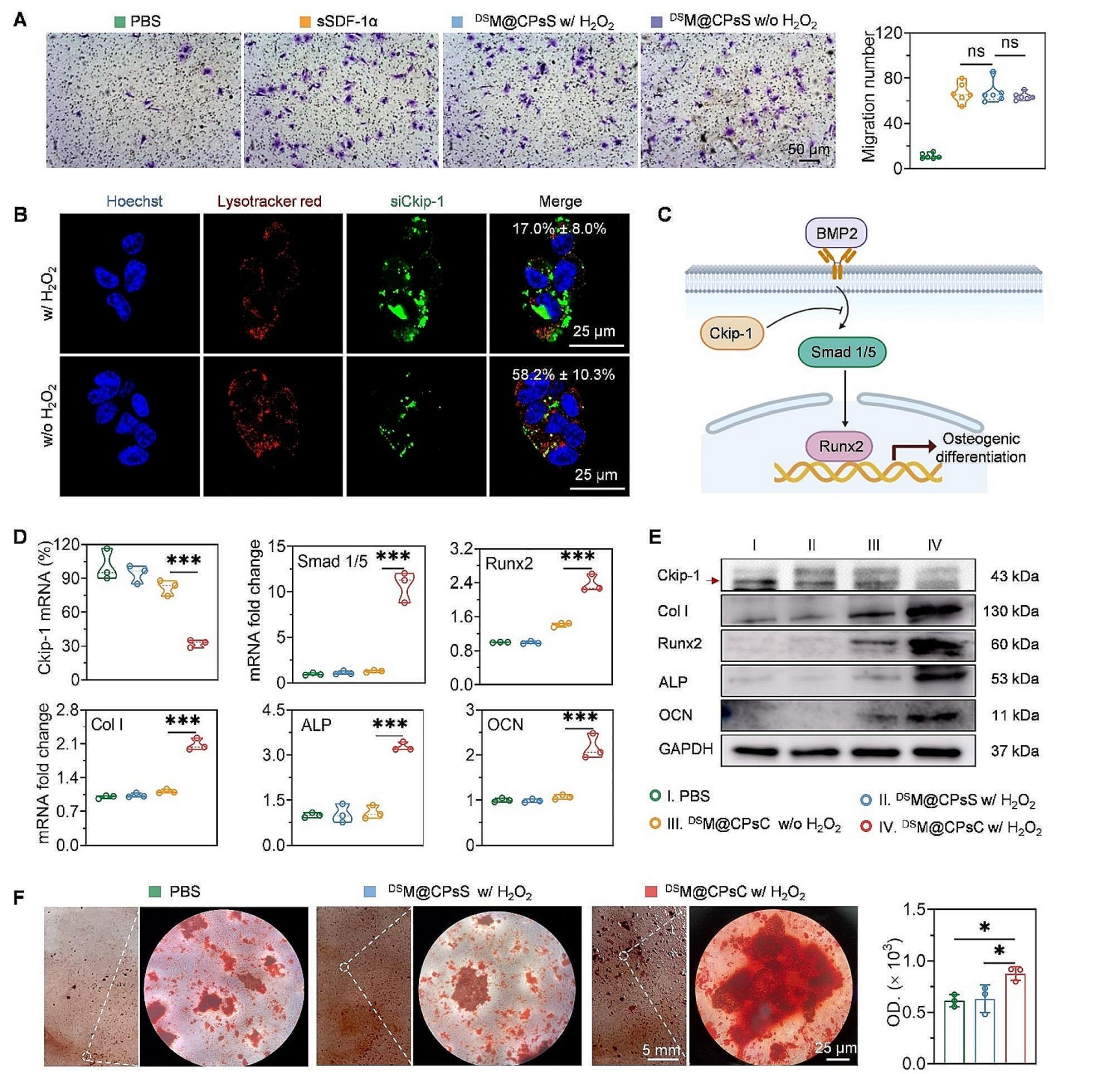
NCs-mediated MSCs migration and osteogenesis in vitro. (**A**) Representative crystal violet-staining images of migrated MSCs and number of migrated MSCs calculated using the ImageJ software (*n* = 6). (**B**) CLSM images of MSCs following 4-h incubation with FAM-siCkip-1-containing ^DS^M@CPsC NCs (1 mg siCkip-1/mL). Cell nuclei and endo/lysosomes were stained with DAPI and Lysotracker Red, respectively. The co-localization ratios were listed (*n* = 10). (**C**) Scheme of the BMP2-Smad 1/5-Runx2 signaling pathway. (**D**) Relative mRNA levels of Ckip-1, Smad 1/5, and osteogenesis-associated genes (Runx2, Col I, ALP, and OCN) in MSCs following NCs treatment (*n* = 3). (**E**) Ckip-1 and various osteogenesis-associated proteins (Runx2, Col 1, ALP, and OCN) levels in MSCs following NCs treatment as resolved by Western blot analysis. (**F**) Representative ARS-staining images of MSCs following NCs treatment and the quantified levels of calcium deposition (*n* = 3)

**Fig. 3:**
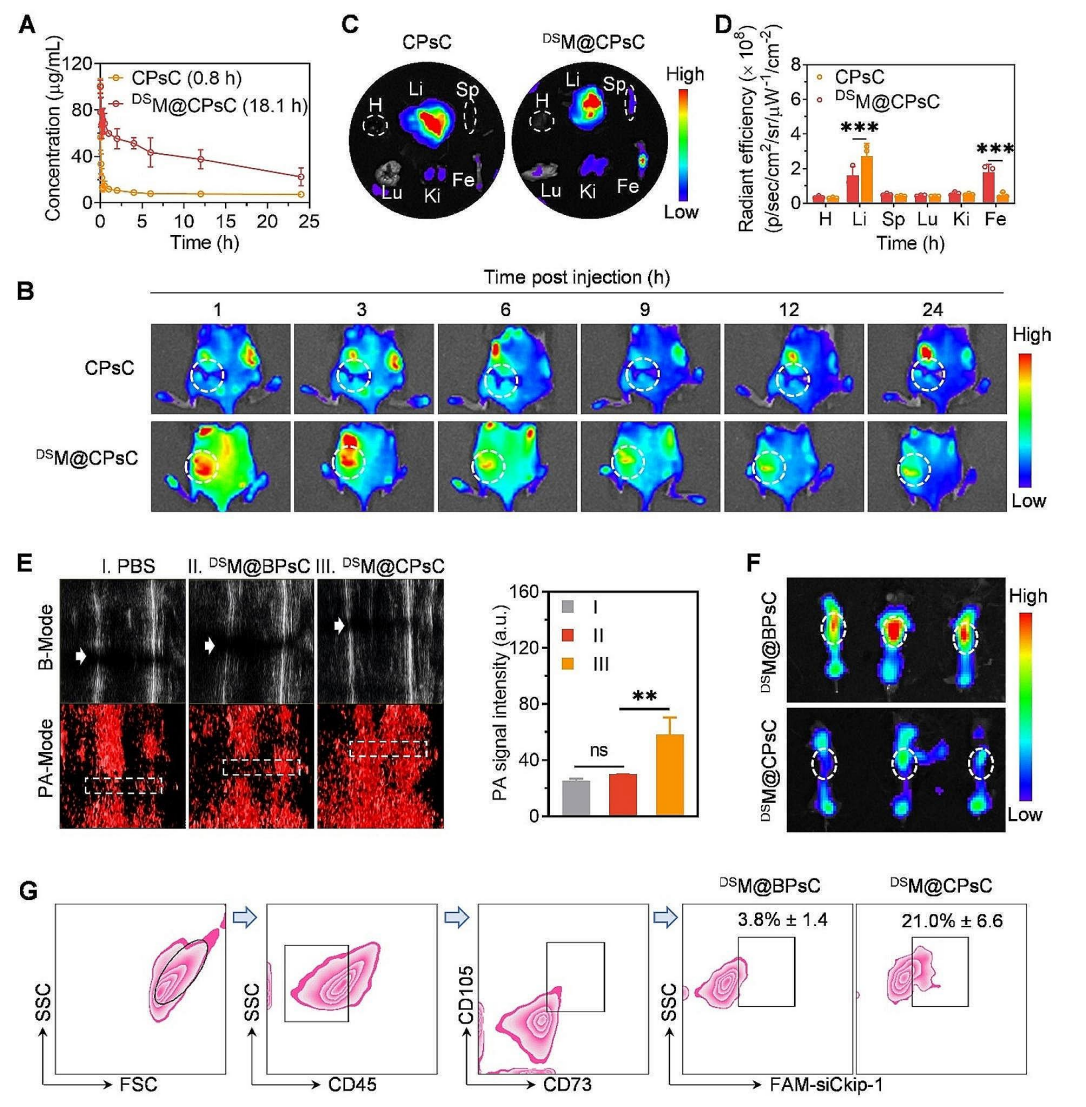
Pharmacokinetics, biodistribution, oxygen generation, membrane shedding, and internalization of NCs by MSCs in vivo. (**A**) Pharmacokinetics of *i.v.* injected Cy5-siCkip-1-containing CPsC NCs and ^DS^M@CPsC NCs in healthy mice (1 mg siCkip-1/kg, *n* = 3). The circulation half-life (t_1/2_) was listed in the parentheses. (**B**) Fluorescence imaging of femur-fractured mice at various time points post *i.v.* injection of Cy5-siCkip-1-containing ^DS^M@CPsC NCs or CPsC NCs (1 mg siCkip-1/kg, *n* = 3). Ex vivo fluorescence imaging (**C**) and calculated fluorescence intensities (**D**) of major organs and femurs isolated from femur-fractured mice at 24 h post *i.v.* injection of Cy5-siCkip-1-containing ^DS^M@CPsC NCs or CPsC NCs (1 mg siCkip-1/kg, *n* = 3). H, Li, Sp, Lu, Ki, and Fe stand for heart, liver, spleen, lung, kidney, and femur, respectively. **E**) Photoacoustic imaging and quantified PA signal intensity of fractured femurs showing the oxygenated hemoglobin level at 3 h post *i.v.* injection of PBS, ^DS^M@BPsC NCs, or ^DS^M@CPsC NCs. The white arrows and rectangles indicate the fractured regions. **F**) Ex vivo fluorescence imaging at the excitation wavelength of 550 nm (for DiI, FRET donor) and the emission wavelength of 670 nm (for Cy5, FRET acceptor) of fractured femurs to evaluate the FRET effect. Femurs were harvested at 6 h post *i.v.* injection of ^DS^M@BPsC NCs or ^DS^M@CPsC NCs comprised of DiI-MM and Cy5-siCkip-1 (1 mg siCkip-1/kg, *n* = 3). The white circles indicate the fractured regions. **G**) FCM plots showing MSCs in the fractured femurs that had internalized NCs at 24 h post *i.v.* injection of FAM-siCkip-1-containing NCs (*n* = 3). MSCs were delineated by the absence of CD45 and presence of CD73 and CD105 (CD45^−^CD73^+^CD105^+^ cells)

**Fig. 4:**
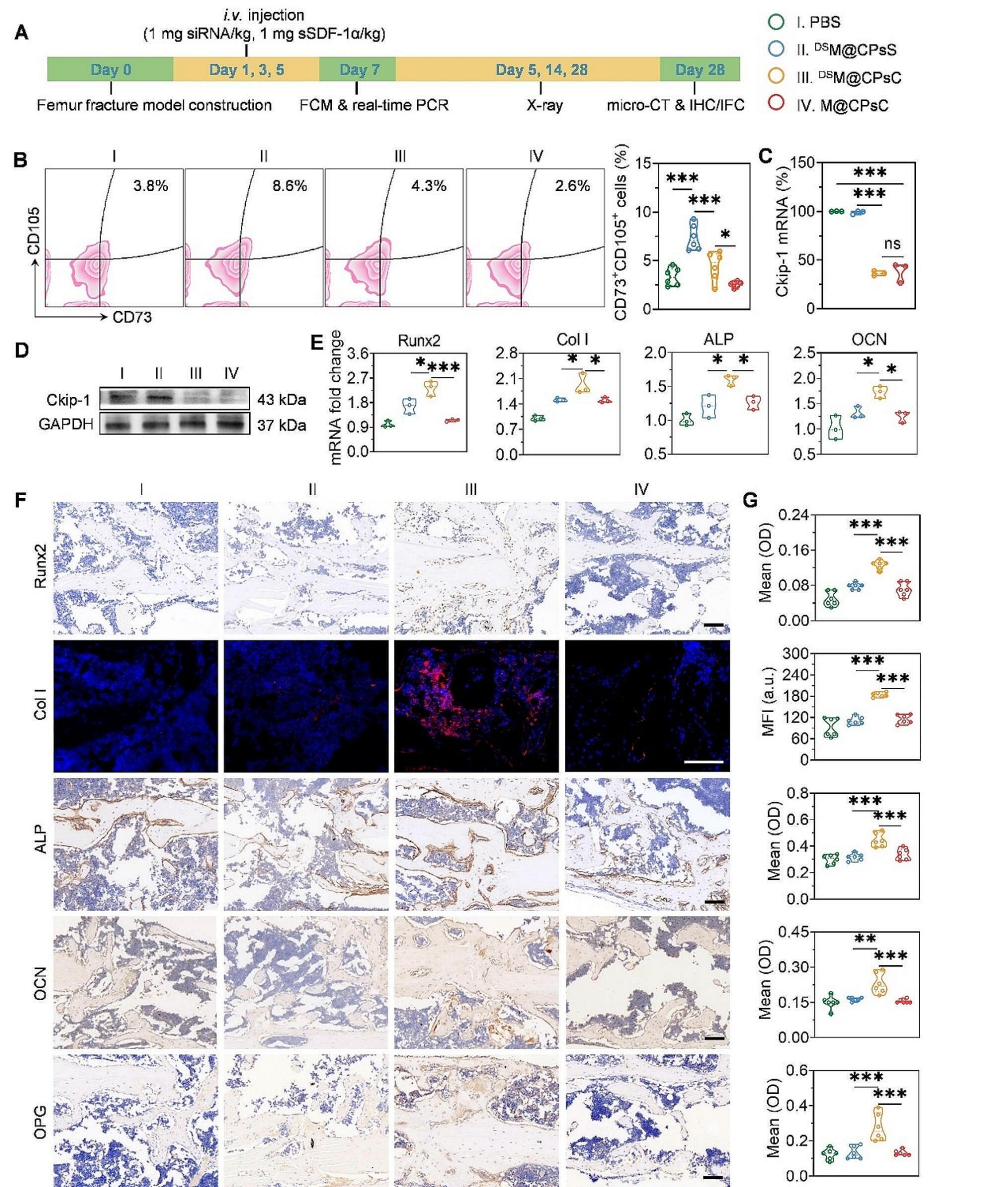
NCs-mediated MSCs recruitment, Ckip-1 silencing, osteogenesis, and mineralization in femur-fractured mice. (**A**) Work flow of the in vivo efficacy study for bone fracture healing. (**B**) FCM plots showing the recruitment of MSCs (CD45^−^CD73^+^CD105^+^ cells) and calculated percentage of MSCs (*n* = 6). Relative Ckip-1 mRNA level (**C**), Ckip-1 protein level (**D**), and mRNA levels of osteogenesis-associated genes (**E**) in femurs harvested from mice at 48 h post the last injection of NCs (*n* = 3). **F**) Histological sections of femur sections stained with anti-Col I, anti-Runx2, anti-ALP, anti-OCN, and anti-OPG on day 28 post femur fracture (scale bar = 100 μm). **G**) Runx2, Col I, ALP, OCN, and OPG expression levels quantified from immune-stained femur sections in (**F**) using the ImageJ software (*n* = 6)

Membrane-cloaked NCs were then constructed using the sonication method [59]. Briefly, MM derived from RAW 264.7 cells was coated onto CPsC NCs (CAT/PG/siCkip-1 = 2.5/10/1, w/w/w) *via* sonication to obtain M@CPsC NCs. Gel electrophoresis revealed retarded siCkip-1 migration at all tested MM/siCkip-1 weight ratios (Supplementary: Figure S5A). At the incremental MM/siCkip-1 weight ratios from 0 to 20, particle size of M@CPsC NCs continuously increased while the zeta potential became more negative. At the MM/siCkip-1 weight ratio of 10, M@CPsC NCs with the diameter of 165 nm and negative zeta potential of -5.2 mV were obtained (Supplementary: Figure S5B, 5 C, and Fig. 1A), more than 90% of MM was coated onto CPsC NCs (Supplementary: Figure S6). Finally, M@CPsC NCs (MM/CAT/PG/siCkip-1 = 10/2.5/10/1, w/w/w/w) were anchored with DS, which was synthesized *via* conjugation of DSPE-PEG_2k_-DBCO and N_3_-sSDF-1α, ultimately forming the ^DS^M@CPsC NCs. Abbreviations and polydispersity index (PDI) for all the NCs were listed in Table S3 and Table S4, respectively. To demonstrate anchoring of DS onto the NCs, the FRET assay was performed [62]. DS and MM were separately labeled with a FRET pair of dyes, Cy3, and DiO, and were then used to construct the ^DS^M@CPsC NCs. When the amount of sSDF-1α was increased, enhanced fluorescence intensity at 585 nm yet weakened fluorescence intensity at 540 nm was observed, indicating the enhanced interaction between the FRET pair due to anchoring of DS onto NCs surfaces (Supplementary: Figure S7). In contrast, such phenomenon was not observed for the mixture of M@CPsC NCs (containing DiO-labeled MM) and Cy3-labeled sSDF-1α (without DSPE as the membrane-anchoring domain) (Supplementary: Figure S8). These results thus demonstrated that DS could be efficiently anchored onto the membrane shell of NCs. At the MM/sSDF-1α weight ratio of 10, ^DS^M@CPsC NCs (MM/CAT/PG/siCkip-1 = 10/2.5/10/1, w/w/w/w) with particle size of ≈ 170 nm and zeta potential of -3.3 mV were obtained (Fig. 1A). The TEM image revealed a clear core–shell structure of ^DS^M@CPsC NCs (Fig. 1B). Western blot analysis further revealed that the characteristic membrane proteins of macrophages (MAC-1 and CD68) were also present on ^DS^M@CPsC NCs (Supplementary: Figure S9), indicating that the membrane-coated NCs could retain the surface proteins inherited from the source cells. After incubation in PBS containing 10% FBS for 6 h, size of the membrane-uncoated NCs (CPsC NCs) dramatically increased, while MM-coated NCs (^DS^M@CPsC NCs) exhibited negligible size alteration (Fig. 1C), indicating that membrane coating could endow NCs with desired serum stability, mainly due to shielding of the positive surface charges of NCs and diminished adsorption of serum proteins.

**Fig. 5:**
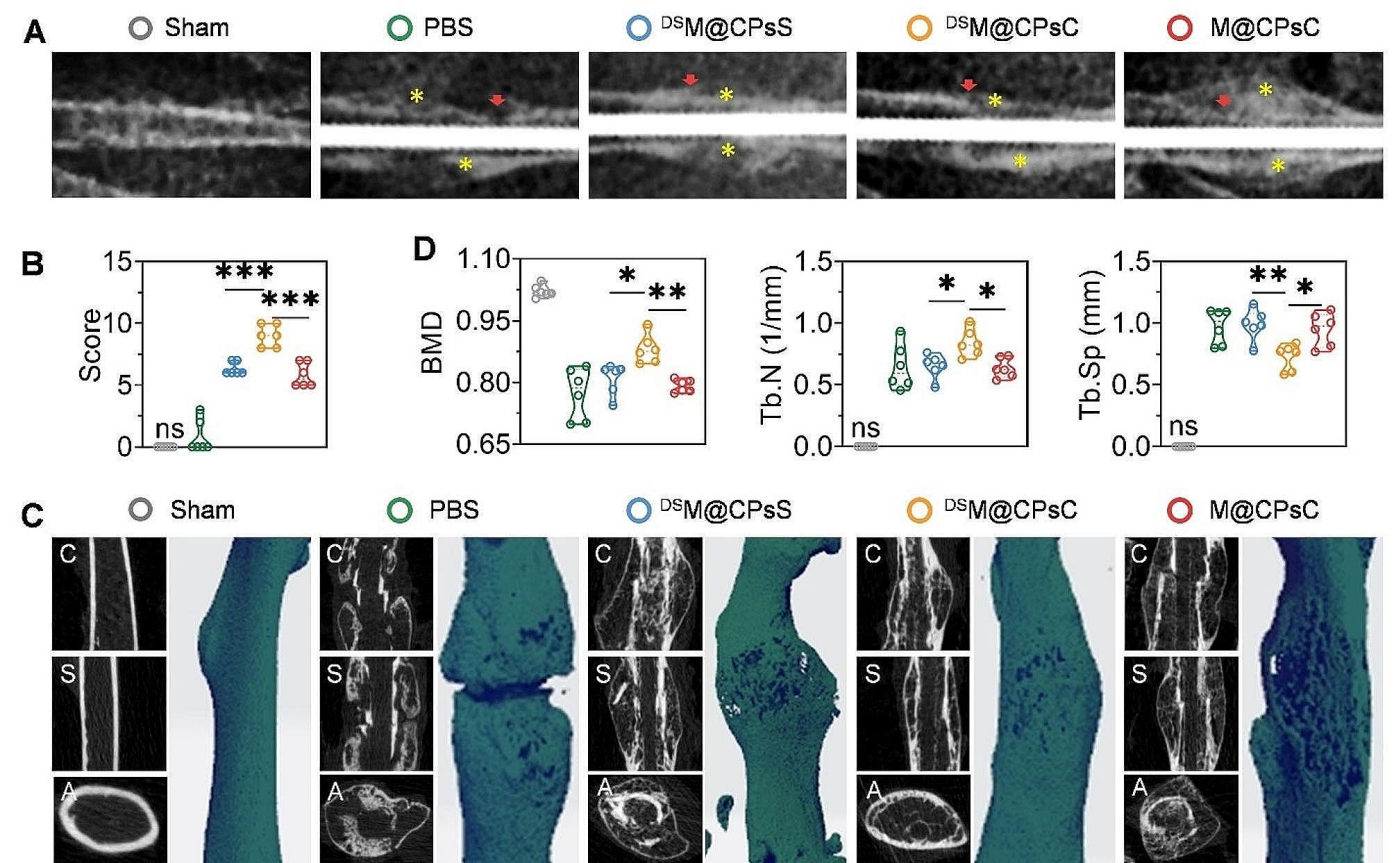
NCs-mediated bone fracture healing in femur-fractured mice. Representative X-ray radiographs (**A**) and total scores calculated from opacity, cortical remodeling and bridging, and periosteal and endosteal reaction in X-ray radiographs (**B**) on day 28 post bone fracture (*n* = 6). The yellow stars and red arrows indicate callus and fractured regions, respectively. Representative reconstructive two-dimensional (left) and three-dimensional (right) micro-CT images (**C**) and mineralization-related parameters (**D**) on day 28 post bone fracture. C and S represent coronal plane and sagittal plane, respectively

**Fig. 6:**
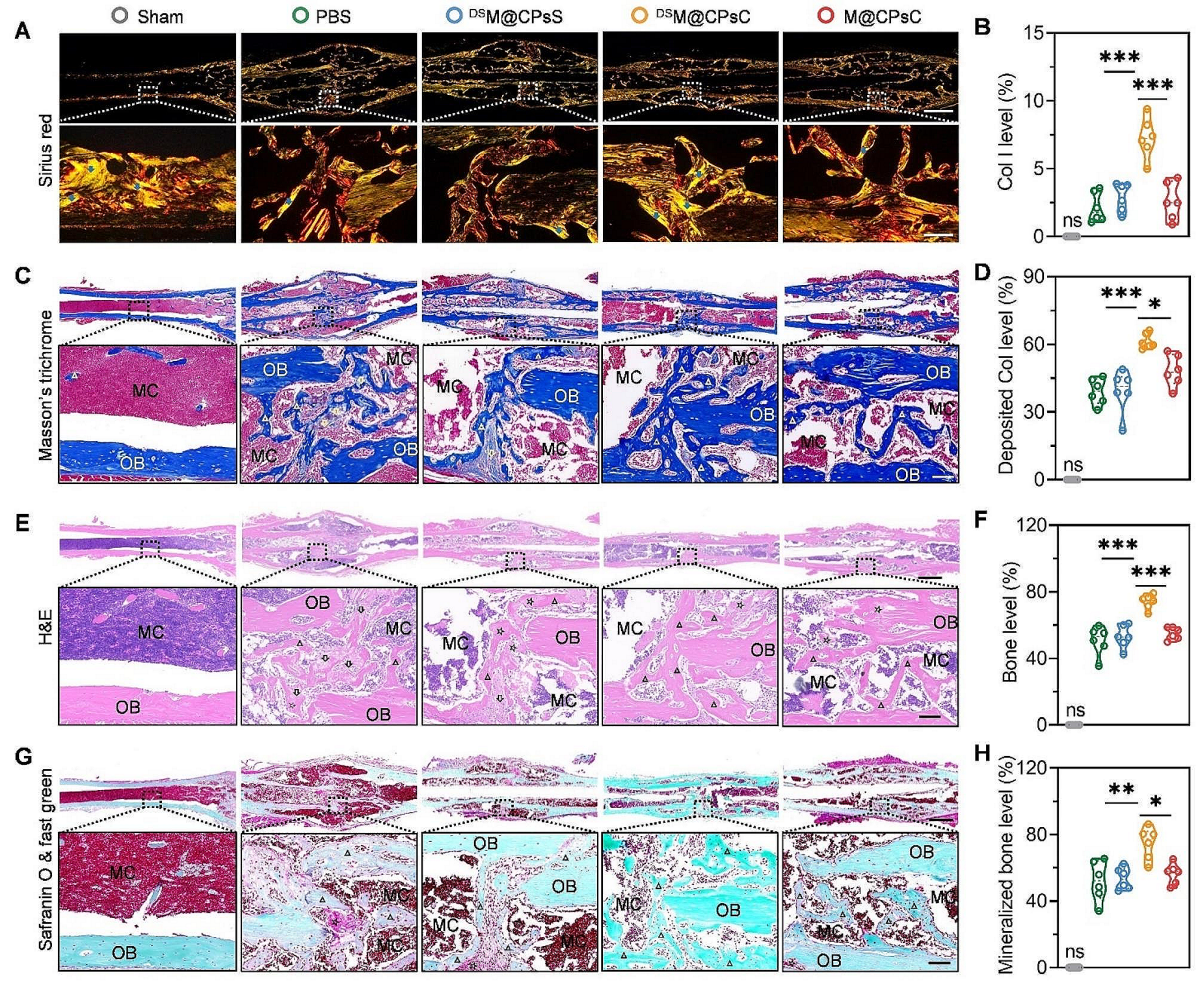
NCs-mediated in vivo osteogenesis. Optical images of femur sections stained with Sirius red (**A**), MT (**C**), H&E (**E**), and Safranin O & fast green (**G**). Femur tissues were harvested on day 28 post fracture. Triangles, stars, black arrows, blue arrows, MC, and OB indicate the lamellar bone, cartilage, fibrous tissue, Col I, marrow cavity, and original bone, respectively. Levels of Col I (**B**), deposited Col (**D**), newly formed bone (**F**), and mineralized bone (**H**) calculated from femur sections in (**A-G**) using the ImageJ software (*n* = 6)

### H_2_O_2_-triggered membrane shedding

CAT-mediated oxygen generation in the presence of H_2_O_2_ was first investigated. Upon treatment with H_2_O_2_ for 1 h, gas bubbles were obviously observed for ^DS^M@CPsC NCs but not ^DS^M@BPsC NCs constructed from bovine serum albumin (BSA) instead of CAT (Supplementary: Figure S10). Then, H_2_O_2_-triggered membrane shedding from ^DS^M@BPsC NCs was investigated in terms of size and zeta potential alteration. As depicted in Fig. 1D, after incubation with H_2_O_2_ (100 µM) for up to 4 h, particle size of ^DS^M@CPsC NCs dramatically increased to ≈ 1000 nm, and the negative zeta potential reversed to positive, indicating shedding of the outer MM shell and exposure of the cationic inner core. In contrast, the H_2_O_2_-nonresponsive ^DS^M@BPsC NCs (lacking the O_2_ generation capability) revealed negligible size and zeta potential alteration upon H_2_O_2_ treatment (Supplementary: Figure S11). The membrane shedding was further verified by the FRET assay conducted on ^DS^M@CPsC NCs consisting of DiI-labeled MM and FAM-siCkip-1 (Fig. 1E). Notably, there was an obvious recovery of FAM fluorescence at 530 nm after 4-h treatment with H_2_O_2_ (100 µM), indicating weakening of the interaction between MM and the ternary NCs. In comparison, the fluorescence spectrum of ^DS^M@BPsC NCs comprised of DiI-labeled MM and FAM-siCkip-1 remained unaltered upon H_2_O_2_ treatment (Supplementary: Figure S12). Such findings were further supported by CLSM observation of ^DS^M@CPsC NCs comprised of DiI-labeled MM and FAM-siCkip-1, which revealed that the colocalized signals of red fluorescence (DiI-MM) and green fluorescence (FAM-siCkip-1) obviously separated from each other upon H_2_O_2_ treatment (100 µM, 4 h, Fig. 1F).

### NCs-mediated MSCs migration and NCs internalization in MSCs in vitro

NCs-mediated migration of MSCs was determined using the transwell migration assay followed by crystal violet staining [65]. As shown in Fig. 2A, large quantities of migrated MSCs were observed after incubation with sSDF-1α, ^DS^M@CPsS NCs, or H_2_O_2_-treated (100 µM, 4 h) ^DS^M@CPsS NCs, indicating that the immobilization of sSDF-1α on NCs had negligible effect on its chemotactic capability.

Then, the internalization of FAM-siCkip-1-containing NCs in MSCs was determined by FCM analysis. MM-cloaked ^DS^M@CPsC NCs was negligibly internalized, mainly because the outer membrane shell prevented the membrane-penetrating helical polypeptide from interacting with the cell membrane. In contrast, H_2_O_2_-treated (100 μm, 4 h) ^DS^M@CPsC NCs could be notably taken up by MSCs, conferring comparable internalization level to the membrane-uncoated CPsC NCs. In consistence, ^DS^M@BPsC NCs lacking the membrane shedding capability still showed low cellular uptake level even after H_2_O_2_ pretreatment (Supplementary: Figure S13). Similar trend was noted when the cellular uptake level was quantitatively determined by spectrofluorimetry (Supplementary: Figure S14). It thus indicated that oxygen-triggered membrane shedding in consequence to CAT-catalyzed H_2_O_2_ decomposition could expose the cationic CPsC NCs, thus mediating robust cytosolic delivery due to the potent membrane activity of the helical polypeptide PG. After internalization into MSCs, ^DS^M@CPsC NCs could effectively avoid the entrapment by endo/lysosomes, as evidenced by the notable separation of green fluorescence (FAM-siCkip-1) from red fluorescence (Lysotracker Red-stained endo/lysosomes) at the low co-localization ratio of ~ 17.0% (Fig. 2B). Moreover, all the tested NCs induced negligible cytotoxicity to MSCs (Supplementary: Figure S15).

### NCs-mediated siCkip-1 gene silencing and osteogenic differentiation in vitro

The siCkip-1 silencing efficiencies of various NCs in MSCs were first determined by real-time PCR. As illustrated in Fig. 2D and Supplementary: Figure S16, H_2_O_2_-treated ^DS^M@CPsC NCs dramatically decreased the Ckip-1 mRNA level by 67.8%, with similar efficiency to CPsC NCs (68.5%) yet remarkably outperforming ^DS^M@CPsC NCs (18.0%), again demonstrating that the outer membrane shell could be removed by oxygen to trigger effective cytosolic delivery and gene silencing. Accordingly, the Ckip-1 protein level was consistently down-regulated by H_2_O_2_-treated ^DS^M@CPsC NCs, as resolved by Western blot analysis (Fig. 2E). Because Ckip-1 could interrupt the Smad 1/5-Runx2 signaling to inhibit osteogenic differentiation (Fig. 2C) [52, 53], the mRNA levels of Smad 1/5 and osteogenesis-associated genes (including Runx2, Col I, ALP, and OCN) in MSCs after NCs treatment were further determined. After incubation with H_2_O_2_-treated ^DS^M@CPsS NCs, the Smad 1/5 and Runx2 mRNA levels were up-regulated by 11.1 and 2.4 folds, respectively (Fig. 2D). As a consequence, the mRNA levels of Col I, ALP, and OCN were also dramatically up-regulated by H_2_O_2_-treated ^DS^M@CPsC NCs, which were 2.1-, 3.3-, and 2.2-fold higher than those of PBS-treated cells, respectively. Consistent results were observed at the protein levels of osteogenesis-associated genes, as determined by Western blot analysis (Fig. 2E). In accordance, MSCs incubated with H_2_O_2_-treated ^DS^M@CPsC NCs revealed obvious red sediments (mineralized nodules) after ARS staining (Fig. 2F), at a significantly higher level than cells incubated with H_2_O_2_-treated ^DS^M@CPsS NCs or PBS. The above results thus demonstrated that upon oxygen-induced membrane shedding, ^DS^M@CPsC NCs could efficiently silence siCkip-1 in MSCs, and thereafter induce osteogenic differentiation *via* activation of the Smad 1/5-Runx2 pathway.

### Pharmacokinetics and in vivo targeting of fractured femur

The pharmacokinetics of Cy5-siCkip-1-containing CPsC NCs and ^DS^M@CPsC NCs in healthy mice after *i.v.* injection was first determined. As shown in Fig. 3A, ^DS^M@CPsC NCs revealed dramatically longer blood circulation time (t_1/2_ = 18.1 h) than CPsC NCs (t_1/2_ = 0.8 h), mainly because cell membrane cloaking could shield the positive surface charges of NCs to diminish protein adhesion and enhance serum stability, thus reducing the clearance by the reticuloendothelial system. The biodistribution of *i.v.* injected Cy5-siCkip-1-containing NCs in femur-fractured mice was further monitored. As shown by whole-animal fluorescence imaging, ^DS^M@CPsC NCs revealed higher fluorescence intensity than CPsC NCs at the fracture region at all tested time points (Fig. 3B and Supplementary: Figure S17), and consistent findings were noted for the ex vivo fluorescence imaging of excised femurs at 24 h post *i.v.* injection (Fig. 3C, D). The enhanced accumulation of ^DS^M@CPsC NCs in the fractured femur could be attributed to their long blood circulation time and the macrophage membrane-assisted inflammation homing. Ex vivo fluorescence imaging of major organs at 24 h post injection revealed accumulation of ^DS^M@CPsC NCs in the liver (Fig. 3C), which may be ascribed to the partial exposure of the inner surface of the cell membrane during the coating process, thus leading to the uptake by reticuloendothelial tissues.

### In vivo oxygen generation, membrane shedding, and internalization of NCs by MSCs

Oxygen generation in the fractured femur was monitored by detecting the echo signals of oxygen using a PA imaging system. As shown in Fig. 3E, obvious echo signals of oxygen were observed in the fractured femur upon administration of the CAT-containing ^DS^M@CPsC NCs but not the BSA-containing ^DS^M@BPsC NCs, indicating that ^DS^M@CPsC NCs could decompose the over-produced H_2_O_2_ in the fractured femur to produce oxygen. The MM shedding from NCs (containing DiI-MM and Cy5-siCkip-1) in vivo was further investigated *via* the FRET assay. At the excitation wavelength of 550 nm for DiI (FRET donor), the fluorescence signal of Cy5 (FRET acceptor, emission wavelength of 670 nm) in the fractured femur at 6 h post *i.v.* injection of ^DS^M@CPsC NCs was significantly lower than that treated with ^DS^M@BPsC NCs (Fig. 3F), indicating decreased FRET effect for ^DS^M@CPsC NCs due to separation of DiI-MM from the Cy5-siCkip-1-containing inner core. Such observation therefore demonstrated that the cloaked MM could effectively shed off from ^DS^M@CPsC NCs but not ^DS^M@BPsC NCs at the fractured site.

As a consequence, FAM-siCkip-1-containing ^DS^M@CPsC NCs could be efficiently internalized by ~ 21% of the MSCs (CD45^−^CD73^+^CD105^+^) in the fractured femur, significantly outperforming the ^DS^M@BPsC NCs (3.8%) that lacked the membrane shedding mechanism (Fig. 3G). Collectively, the above results indicated that upon MM-assisted accumulation to the fractured femur, the ^DS^M@CPsC NCs could decompose the local H_2_O_2_ to generate oxygen and drive the shedding of the outer membrane shell, thereby exposing the cationic inner core to enable efficient delivery into MSCs.

### NCs-mediated MSCs recruitment, Ckip-1 silencing, and osteogenesis in fractured femur

The quantities of MSCs in the fractured bones and their osteogenic differentiation are vital for fracture healing [22]. Therefore, we first determined the percentage of MSCs in the fractured femur of mice after NCs administration (Fig. 4A, B). Compared with PBS, ^DS^M@CPsS NCs significantly elevated the percentage of MSCs (CD45^−^CD73^+^CD105^+^) from 3.8 to 8.6%, which nonetheless, was decreased to 2.6% by M@CPsC NCs. The increment of MSCs by ^DS^M@CPsS NCs demonstrated that the anchored sSDF-1α could recruit MSCs to the fractured site, while the decrease of MSCs by M@CPsC NCs could be explained by the siCkip-1-mediated osteogenic differentiation of MSCs. Due to such a counteracting effect, ^DS^M@CPsC NCs led to significantly lower MSCs level (4.3%) than ^DS^M@CPsS NCs yet higher MSCs level than M@CPsC NCs.

Then, the Ckip-1 mRNA and protein levels in the fractured femur after *i.v.* injection of NCs were determined by real-time PCR and Western blot, respectively. As illustrated in Fig. 4C, the Ckip-1 mRNA level was down-regulated by 61.8% and 63.5% after administration with M@CPsC NCs and ^DS^M@CPsC NCs, respectively, and the Ckip-1 protein was consistently down-regulated (Fig. 4D). As a consequence, ^DS^M@CPsC NCs significantly outperformed ^DS^M@CPsS NCs and M@CPsC NCs to remarkably elevate the mRNA levels as well as protein levels of osteogenesis-associated genes (Runx2, Col I, ALP, and OCN), as determined by real-time PCR (Fig. 4E) and immunostaining (Fig. 4F, G). Consistently, ^DS^M@CPsC NCs also notably elevated the osteoprotegerin (OPG) level by 2.2 folds (Fig. 4F, G). The above results collectively demonstrated that the sSDF-1α-mediated MSCs recruitment could cooperate with siCkip-1-mediated osteogenic differentiation to facilitate bone formation.

### NCs-mediated fracture healing

The capabilities of *i.v.* injected NCs in fostering fracture healing was evaluated in femur-fractured C57BL/6 mice using both X-ray and micro-computed tomography (micro-CT) examination. As shown in Fig. 5A and Supplementary: Figure S18, mice treated with ^DS^M@CPsC NCs showed calcified callus on day 14 post fracture, and the newly formed bone had bridged the fracture gap on both day 14 and 28 post fracture. Comparatively, mice treated with PBS revealed negligible bone bridging and low apparent radiographic density of callus even on day 28 post fracture. For mice treated with ^DS^M@CPsS NCs and M@CPsC NCs, only partially bridged fracture gap with moderate calcified callus was noted on day 28. Such observation indicated that ^DS^M@CPsC NCs could facilitate the formation of functional callus to accelerate fracture healing. Then, the fracture healing was scored based on the opacity, cortical remodeling and bridging, periosteal and endosteal reaction in X-ray radiographs on day 28 (Fig. 5B and Supplementary: Figure S19). Mice treated with ^DS^M@CPsC NCs conferred the highest total scores, significantly higher than those treated with ^DS^M@CPsS NCs or M@CPsC NCs. Consistent results were observed for micro-CT imaging (Fig. 5C), wherein ^DS^M@CPsC NCs provoked the highest level of mineralization of the fracture callus, significantly outperforming ^DS^M@CPsS NCs and M@CPsC NCs. In particular, ^DS^M@CPsC NCs remarkably elevated the BMD and Tb.N by 1.1 and 1.3 folds, respectively, while decreased the Tb.Sp in femurs by 25.2% (Fig. 5D). The above findings thus collectively substantiated the pronounced capability of ^DS^M@CPsC NCs in promoting fracture healing, based on the combined contribution of sSDF-1α-mediated recruitment of MSCs and siCkip-1-mediated osteogenic differentiation that facilitated the formation and mineralization of functional callus.

During fracture healing, Col I expression, total collagen deposition, new bone generation, and bone mineralization are critical steps in the formation of functional callus and subsequently callus remodeling [1]. Therefore, the levels of Col I, deposited total collagen, newly formed bone, and mineralized bone were further monitored using Sirius red, MT, H&E, and Safranin O & fast green staining, respectively. As shown in Fig. 6A, B, more Col I (characterized with closely-arranged and organized red/yellow fibrous tissue with bright bi-refraction) was observed in Sirius red-stained cross-sections of femurs after treatment with ^DS^M@CPsC NCs. Accordingly, ^DS^M@CPsC NCs led to pronounced new bone formation, with the total deposited collagen content and bone level being 1.6 and 1.5 folds higher than those in PBS-treated femurs, respectively (Fig. 6C-F). Moreover, ^DS^M@CPsC NCs provoked notable bone mineralization as shown by Safranin O & fast green staining (green), significantly outperforming M@CPsC NCs and ^DS^M@CPsS NCs (Fig. 6G, H). These results again demonstrated that treatment with ^DS^M@CPsC NCs could accelerate the formation and mineralization of callus to facilitate fracture healing.

### Biocompatibility of NCs

The systemic toxicity of ^DS^M@CPsC NCs following *i.v.* injection was explored in healthy mice. H&E-stained major organ sections revealed lack of obvious pathological abnormalities (Supplementary: Figure S20). Moreover, no abnormality was observed in all tested hematological and biochemical parameters (Supplementary: Figure S21, S22), which demonstrated the desired biocompatibility of ^DS^M@CPsC NCs, mainly attributed to the cell membrane cloaking that shielded the cationic charges of the inner NCs.

## Conclusion

MM-cloaked NCs featuring oxygen-triggered membrane shedding were developed for the fracture-targeted and hierarchical co-delivery of sSDF-1α and siCkip-1, fostering effective bone fracture healing by recruiting endogenous MSCs and enhancing their osteogenic differentiation. The outer MM shell allowed targeted accumulation of NCs to the fracture lesions, while CAT inside the NCs decomposed the over-produced H_2_O_2_ in the inflamed microenvironment to generate oxygen bubbles and shed off the DS-anchored MM, thus exposing the helical polypeptide-constituted inner core to aid trans-membrane siCkip-1 delivery into MSCs. Hence, sSDF-1α-guided MSCs recruitment cooperated with siCkip-1-mediated osteogenic differentiation to promote bone fracture healing. This study provides an enlightened strategy to overcome the multiple physiological barriers against the hierarchical co-delivery of drug cargoes into different cellular compartments. The mechanism of concurrently managing MSCs recruitment and differentiation may also render a promising paradigm for bone fracture repair. While varieties of biocompatible nanocarriers have been developed for targeted drug delivery [66–68], the current nanocomplexes afford the advantages of synchronous delivery yet asynchronous release of peptide and siRNA cargoes, contributing to the cooperative regulation of the microenvironment at the fractured site. Compared to drug-releasing scaffolds that are widely explored for the management of surgery-accessible bone defects/fractures, the nano-delivery system capable of bone fracture targeting and fostering bone fracture healing may feature more benefits for slight fracture management, thus diversifying the therapeutic options for bone fracture.

### Electronic supplementary material

Below is the link to the electronic supplementary material.

Supplementary Material 1

Supplementary Material 2

## Data Availability

All data required in this study are included in the manuscript and the supplementary materials.

## Abbreviations

MSCs: Mesenchymal stem cells
NCs: Nanocomplexes
MM: Macrophage membrane
PG: Guanidine-rich, α-helical polypeptide
H_2_O_2_: Hydrogen peroxide
sSDF-1α: Short stromal derived factor-1α peptide
OCN: Osteocalcin
OPN: Osteopontin
ALP: Alkaline phosphatase
Col I: Type I collagen
ROS: Reactive oxygen species
siRNA: Small interfering RNA
CAT: Catalase
DSPE: 1,2-Dioleoyl-sn-glycero-3-phosphoethanolamine
DSPE-PEG_2k_-DBCO: 1,2-Dioleoyl-sn-glycero-3-phosphoethanolamine-conjugated diphenyl-poly(ethylene glycol)_2k_ monomethyl ether-cyclooctyne
MTT: 3-(4,5-Dimethyl-2-thiazolyl)-2,5-diphenyl tetrazolium bromide
DAPI: 4’,6-Diamidino-2-phenylindole
siScr: siRNA with scrambled sequence
FBS: Fetal bovine serum
FRET: Fluorescence resonance energy transfer
HPLC: High performance liquid chromatography
BSA: Bovine serum albumin
DMSO: Dimethyl sulfoxide
IFC: Immunofluorescence
IHC: Immunohistochemical
ROI: Region of interest
BMD: Bone mineral density
Tb.N: trabecular number
Tb.Sp: Trabecular separation/spacing
MT: Masson’s trichrome
H&E: haematoxylin and eosin
CLSM: Confocal laser scanning microscopy
TEM: Transmission electron microscopy
FCM: Flow cytometric
