# Supplementary material for: Macrophage membrane-reversibly camouflaged nanotherapeutics accelerate fracture healing by fostering MSCs recruitment and osteogenic differentiation
Source: J Nanobiotechnology. 2024 Jul 12;22:411. doi: 10.1186/s12951-024-02679-y (PMC11241938; doi:10.1186/s12951-024-02679-y)
Supplement: Supplementary file 1 — Supplementary Material 1 [file 12951_2024_2679_MOESM1_ESM.pdf]

Supplementary Information

# Macrophage membrane-reversibly camouflaged nanotherapeutics accelerate fracture healing by fostering MSCs recruitment and osteogenic differentiation

*Cheng Wu, Jing Yan, Chenglong Ge, Lucheng Xie, Yunjie He, Ziyin Zhao, Yekun Deng, Qirong Dong*, and Lichen Yin**

**Table S1**. Sequences of siRNA.

| Name | Sequences |
|---|---|
| siCkip-1, sense | 5’-CUACUCGAGACAGAGCAAATT-3’ |
| siCkip-1, anti-sense | 5’-UUUGCUCUGUCUCGAGUAGTT-3’ |
| siScr, sense | 5’-UUCUCCGAACGUGUCACGUTT-3’ |
| siScr, anti-sense | 5’-ACGUGACACGUUCGGAGAATT-3’ |

**Table S2**. Sequences of forward (F) and reverse (R) primers.

| Name | Sequences |
|---|---|
| Ckip-1 (F) | 5’-CCATCCCAAGTTCAGTGGCAT-3’ |
| Ckip-1 (R) | 5’-GAAGCGAATGGTGTCTTCCCG-3’ |
| ALP (F) | 5’-CAAGGACCAACTACACCA-3’ |
| ALP (R) | 5’-AGGGAAGGGTCAGTCAGGTT-3’ |
| Collagen I (F) | 5’-CAATGGTGAGACGTGGAAAC-3’ |
| Collagen I (R) | 5’-GTTGGGACAGTCCAGTTCT-3’ |
| Runx2 (F) | 5’-GCAGAAAGATGCTTCCGGTC-3’ |
| Runx2 (R) | 5’-TCAACAACAGGGAACTGCCA-3’ |
| OCN (F) | 5’-GGAGGGCAGTAAGGTGGTGA-3’ |
| OCN (F) | 5’-GAAGCCAATGTGGTCCGC-3’ |
| Smad 1/5 (F) | 5’-CAGGCAGTTGCTTACGAGGA-3’ |
| Smad 1/5 (R) | 5’-AAAGGTGGACTCCTTTCCCA-3’ |
| GAPDH (F) | 5’-GAAGGTGAAGGTCGGAGTC-3’ |
| GAPDH (R) | 5’-GAAGATGGTGATGGGATTTC-3’ |

**Table S3**. Full names and abbreviations of NCs.

| Abbreviation | Full name |
|---|---|
| PsS NCs | PG/siScr nanocomplexes |
| PsC NCs | PG/siCkip-1 nanocomplexes |
| CPsC NCs | CAT/PG/siCkip-1 nanocomplexes |
| M@CPsC NCs | macrophage membrane-cloaked CPsC NCs |
| M@CPsS NCs | macrophage membrane-cloaked CPsS NCs |
| M@BPsC NCs | macrophage membrane-cloaked BSA/PG/siCkip-1 NCs |
| $^{DS}$M@CPsC NCs | DSPE-PEG$_{2k}$-sSDF-1α-anchored M@CPsC NCs |
| $^{DS}$M@BPsC NCs | DSPE-PEG$_{2k}$-sSDF-1α-anchored M@BPsC NCs |
| $^{DS}$M@CPsS NCs | DSPE-PEG2k-sSDF-1α-anchored M@CPsS NCs |

**Table S4**. Size and PDI of all NCs (*n* = 3).

| NCs | Size | PDI |
|---|---|---|
| PsC | 116.1 ± 8.0 | 0.26 ± 0.07 |
| CPsC | 126.1 ± 2.7 | 0.23 ± 0.02 |
| M@CPsC | 164.5 ± 4.6 | 0.18 ± 0.06 |
| $^{DS}$M@CPsC | 170.8 ± 6.3 | 0.22 ± 0.05 |

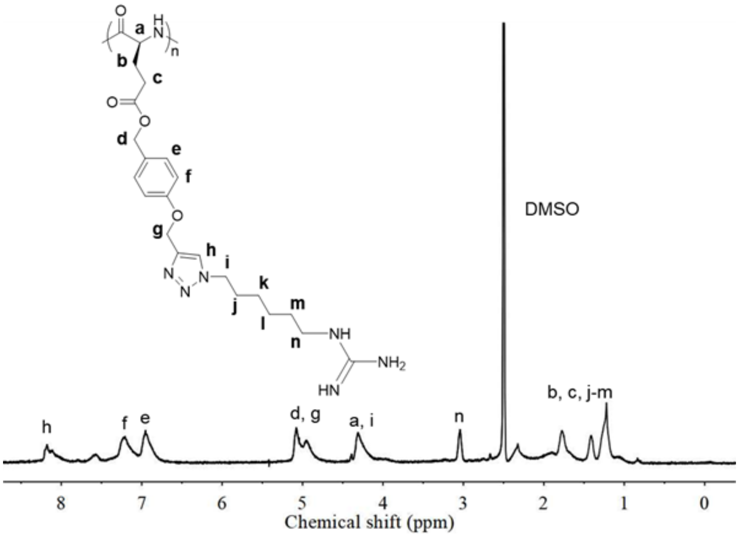


**Figure S1**. $^{1}$H NMR spectrum of PG in DMSO-$d_6$.

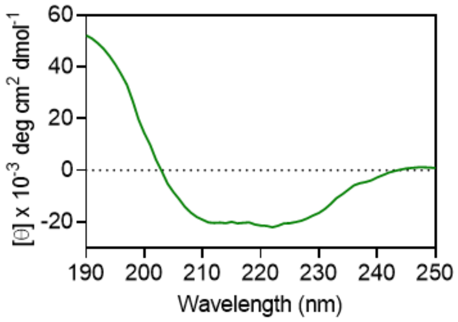


**Figure S2**. CD spectrum of PG in deionized water (0.2 mg/mL).

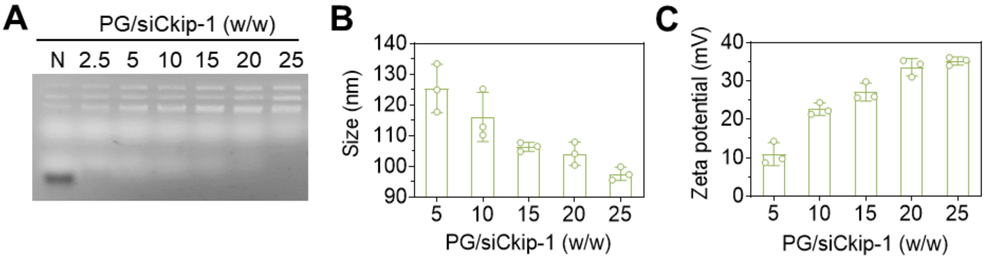


**Figure S3.** Agarose gel electrophoresis image (A), size (B) and zeta potential (C) of PsC NCs at various PG/siCkip-1 weight ratios (*n* = 3).

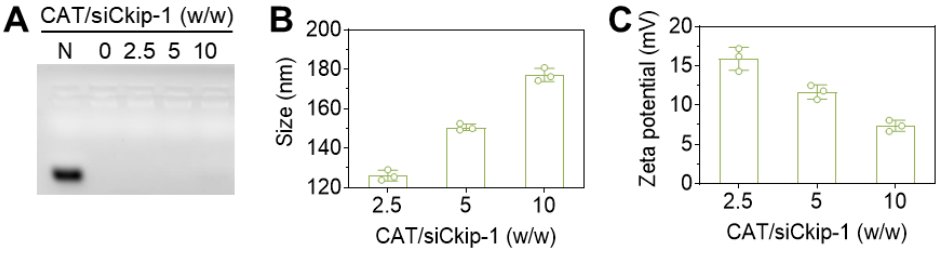


**Figure S4.** Agarose gel electrophoresis image (A), size (B), and zeta potential (C) of CPsC NCs at various CAT/siCkip-1 weight ratios (PG/siCkip-1 = 10, w/w, *n* = 3).

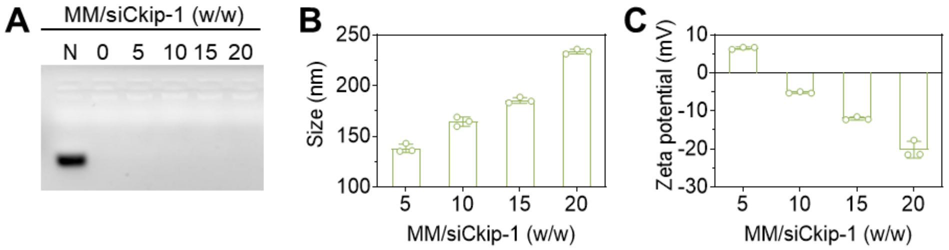


**Figure S5.** Agarose gel electrophoresis image (A), size (B), and zeta potential (C) of M@CPsC NCs at various MM/siCkip-1 weight ratios (CAT/siCkip-1 = 2.5, PG/siCkip-1 =10, w/w, *n* = 3).

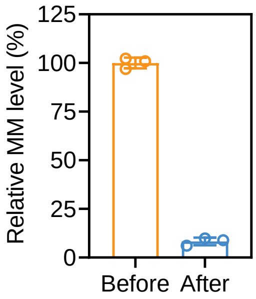


**Figure S6.** Relative MM levels of M@CPsC NCs (MM/CAT/PG/siCkip-1 = 10/2.5/10/1, w/w/w/w) composed of DiD-stained MM before or after centrifugation (10,000 g, 10 min) (*n* = 3).

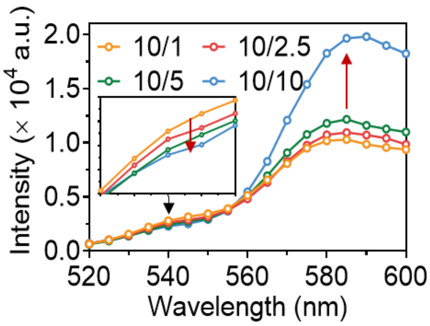


**Figure S7.** Fluorescence emission spectra ($\lambda_{ex}$ = 480 nm) of $^{DS}$M@CPsC NCs composed of DiO-stained MM and Cy3-labeled, DSPE-PEG$_{2k}$-conjugated sSDF-1α (Cy3-DS) at various membrane protein/sSDF-1α weight ratios. Black arrow points to the enlarged area.

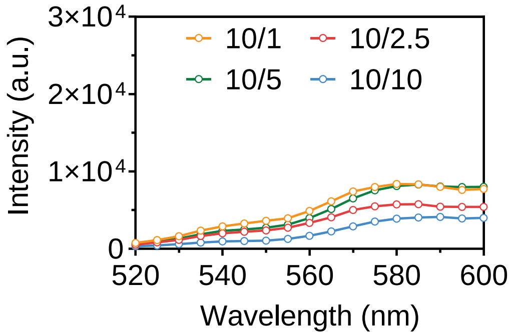


**Figure S8.** Fluorescence emission spectra ($\lambda_{ex}$ = 480 nm) of the mixture of M@CPsC NCs (containing DiO-stained MM) and Cy3-labeled sSDF-1α (without DSPE as the membrane-anchoring domain) at various membrane protein/sSDF-1α weight ratios.

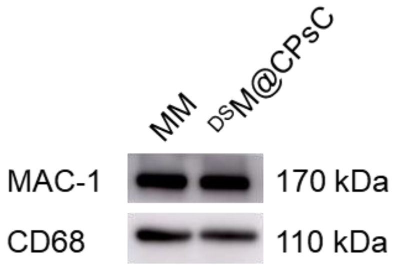


**Figure S9.** Western blot analysis of characteristic membrane markers of macrophages (MAC-1, CD68) in the isolated macrophage membrane (MM) and $^{DS}$M@CPsC NCs.

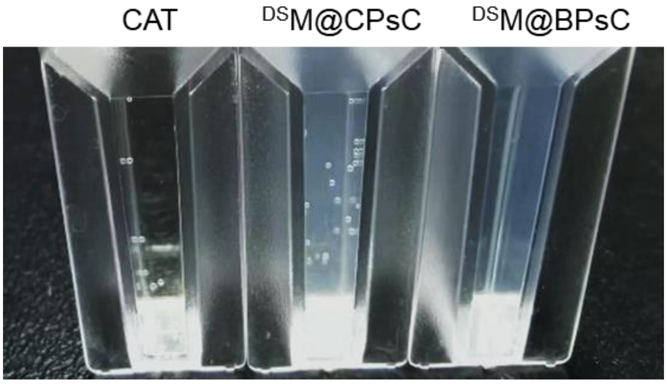


**Figure S10.** Photographs of CAT, $^{DS}$M@CPsC NCs, and $^{DS}$M@BPsC NCs (0.1 mg CAT or BSA/mL) after incubation with $H_2O_2$ (50 mM) for 1 h.

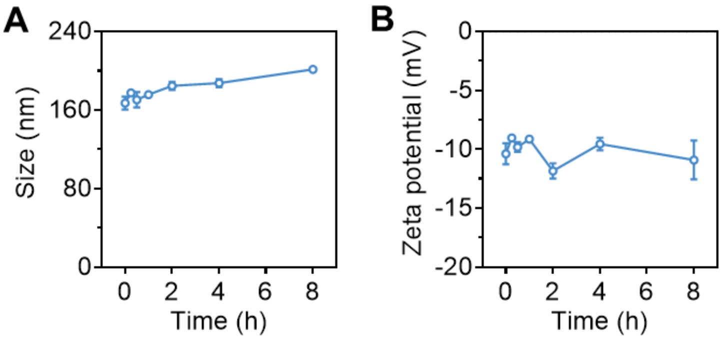


**Figure S11.** Size (a) and zeta potential (b) of $^{DS}$M@BPsC NCs after treatment with $H_2O_2$ (100 μM) for various time ($n$ = 3).

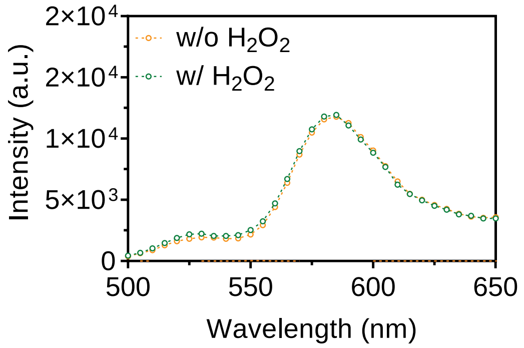


**Figure S12.** Fluorescence emission spectra ($\lambda_{ex}$ = 494 nm) of FAM-siCkip-1-containing $^{DS}$M@BPsC NCs with (w/) or without (w/o) $H_2O_2$ treatment (100 μM, 4 h). MM was pre-stained with DiI.

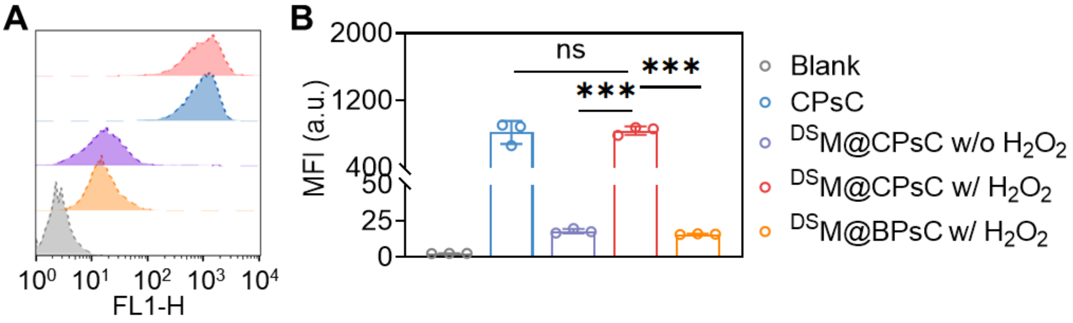


**Figure S13.** FCM histograms and mean fluorescence intensity of MSCs after 4-h incubation with various FAM-siCkip-1-containing NCs ($n$ = 3).

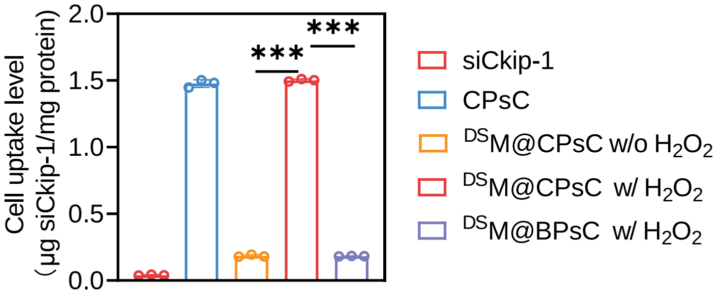


**Figure S14.** Uptake levels of various NCs containing FAM-siCkip-1 (1 μg siCkip-1/mL) in MSCs after incubation for 4 h (*n* = 3).

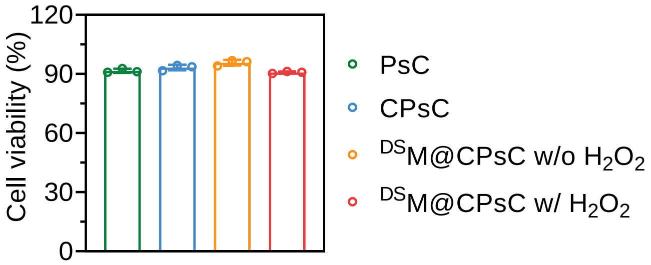


**Figure S15.** Cytotoxicity of PsC NCs, CPsC NCs, $^{DS}$M@CPsC NCs, or $H_2O_2$ (100 μM, 4 h)-pretreated $^{DS}$M@CPsC NCs in MSCs following 24-h incubation (*n* = 3).

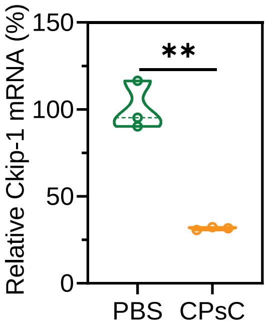


**Figure S16.** Ckip-1 mRNA levels in MSCs following CPsC NCs treatment (*n* = 3).

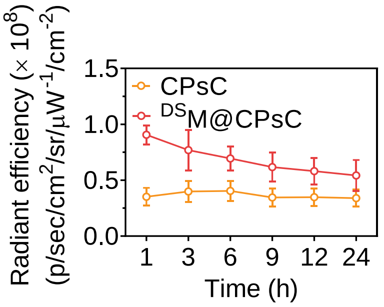


**Figure S17.** Calculated fluorescence intensities in the circled fracture regions in Figure 3b at various time points post *i.v.* injection of Cy5-siCkip-1-containing $^{DS}$M@CPsC NCs or CPsC NCs (1 mg siCkip-1/kg, $n$ = 3).

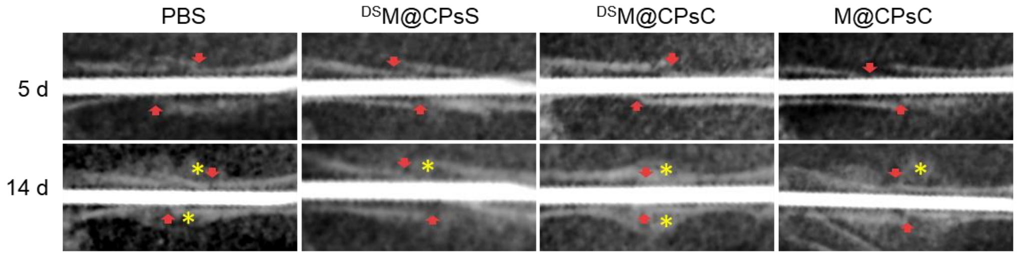


**Figure S18.** Representative X-ray radiographs of mice on day 5 and 14 post femur fracture. Stars represent callus, and red arrows indicate the fractured regions.

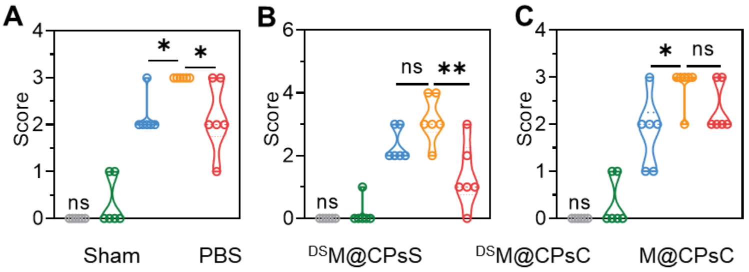


**Figure S19.** The scores of opacity (A), cortical remodeling and bridging (B), and periosteal and endosteal reaction (C) assessed from radiographic images on day 28 ($n$ = 6).

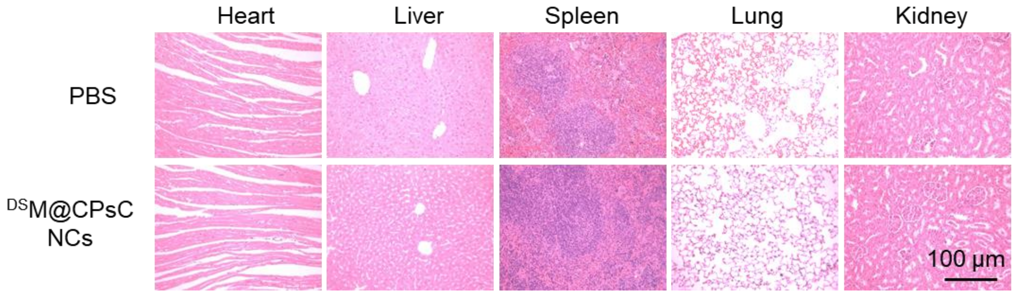


**Figure S20.** H&E staining of major organ sections (1 mg siCkip-1/kg, $n$ = 3).

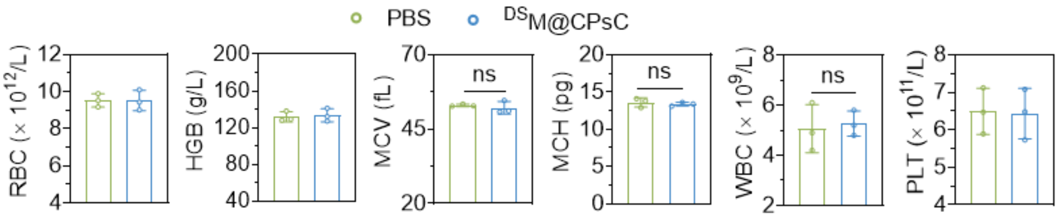


**Figure S21.** Hematological parameters of mice receiving three *i.v.* injections of PBS or $^{DS}$M@CPsC NCs (1 mg siCkip-1/kg, $n$ = 3).

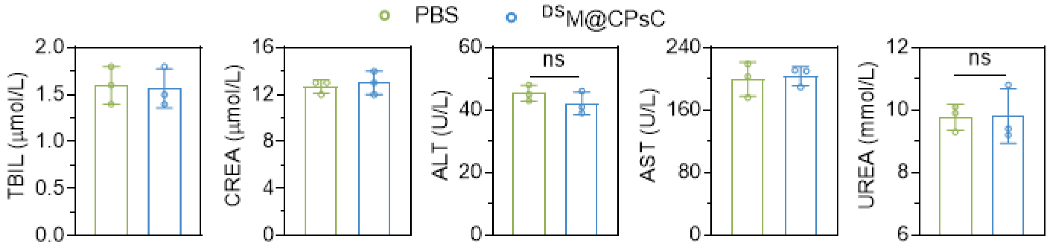


**Figure S22**. Serum biochemical levels of mice receiving three *i.v.* injections of PBS or $^{DS}$M@CPsC NCs (1 mg siCkip-1/kg, $n$ = 3).
