# Supplementary material for: Macrophage membrane-reversibly camouflaged nanotherapeutics accelerate fracture healing by fostering MSCs recruitment and osteogenic differentiation
Source: J Nanobiotechnology. 2024 Jul 12;22:411. doi: 10.1186/s12951-024-02679-y (PMC11241938; doi:10.1186/s12951-024-02679-y)
Supplement: Supplementary file 2 — Supplementary Material 2 [file 12951_2024_2679_MOESM2_ESM.pdf]

Supplementary Information

Macrophage membrane-reversibly camouflaged nanotherapeutics accelerate fracture healing by fostering MSCs recruitment and osteogenic differentiation

Cheng Wu, Jing Yan, Chenglong Ge, Lucheng Xie, Yunjie He, Ziyin Zhao, Yekun Deng [a]*, Qirong Dong*, and Lichen Yin**

Table S1. Sequences of siRNA.

Name	Sequences
siCkip-1, sense	5’-CUACUCGAGACAGAGCAAATT-3’
siCkip-1, anti-sense	5’-UUUGCUCUGUCUCGAGUAGTT-3’
siScr, sense	5’-UUCUCCGAACGUGUCACGUTT-3’
siScr, anti-sense	5’-ACGUGACACGUUCGGAGAATT-3’

Table S2. Sequences of forward (F) and reverse (R) primers.

Name	Sequences
Ckip-1 (F)	5’-CCATCCCAAGTTCAGTGGCAT-3’
Ckip-1 (R)	5’-GAAGCGAATGGTGTCTTCCCG-3’
ALP (F)	5’-CAAGGACCAACTACACCA-3’
ALP (R)	5’-AGGGAAGGGTCAGTCAGGTT-3’
Collagen I (F)	5’-CAATGGTGAGACGTGGAAAC-3’
Collagen I (R)	5’-GTTGGGACAGTCCAGTTCT-3’
Runx2 (F)	5’-GCAGAAAGATGCTTCCGGTC-3’
Runx2 (R)	5’-TCAACAACAGGGAACTGCCA-3’
OCN (F)	5’-GGAGGGCAGTAAGGTGGTGA-3’
OCN (F)	5’-GAAGCCAATGTGGTCCGC-3’
Smad 1/5 (F)	5’-CAGGCAGTTGCTTACGAGGA-3’
Smad 1/5 (R)	5’-AAAGGTGGACTCCTTTCCCA-3’
GAPDH (F)	5’-GAAGGTGAAGGTCGGAGTC-3’
GAPDH (R)	5’-GAAGATGGTGATGGGATTTC-3’

Table S3. Full names and abbreviations of NCs.

**Abbreviation**	**Full name**
PsS NCs	PG/siScr nanocomplexes
PsC NCs	PG/siCkip-1 nanocomplexes
CPsC NCs	CAT/PG/siCkip-1 nanocomplexes
M@CPsC NCs	macrophage membrane-cloaked CPsC NCs
M@CPsS NCs	macrophage membrane-cloaked CPsS NCs
M@BPsC NCs	macrophage membrane-cloaked BSA/PG/siCkip-1 NCs
^{DS}M@CPsC NCs	DSPE-PEG$_{2k}$-sSDF-1α-anchored M@CPsC NCs
^{DS}M@BPsC NCs	DSPE-PEG$_{2k}$-sSDF-1α-anchored M@BPsC NCs
^{DS}M@CPsS NCs	DSPE-PEG2k-sSDF-1α-anchored M@CPsS NCs

Table S4. Size and PDI of all NCs (*n* = 3).

NCs	Size	PDI
PsC	116.1 ± 8.0	0.26 ± 0.07
CPsC	126.1 ± 2.7	0.23 ± 0.02
M@CPsC	164.5 ± 4.6	0.18 ± 0.06
^{DS}M@CPsC	170.8 ± 6.3	0.22 ± 0.05

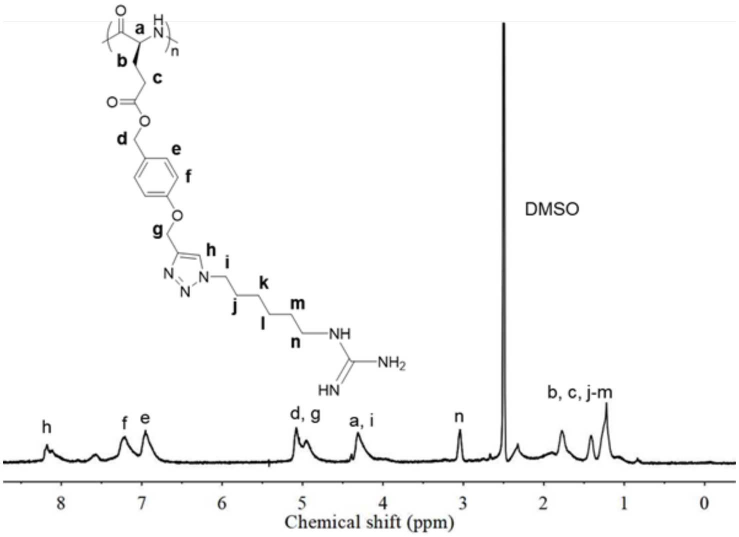

Figure S1. ^{1}H NMR spectrum of PG in DMSO-d_6.

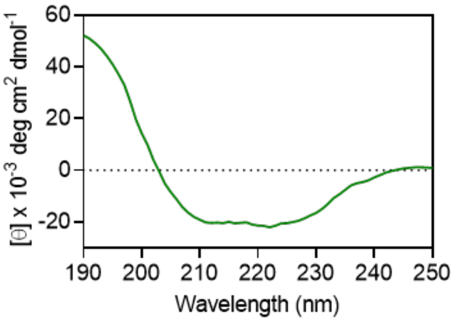

Figure S2. CD spectrum of PG in deionized water (0.2 mg/mL).

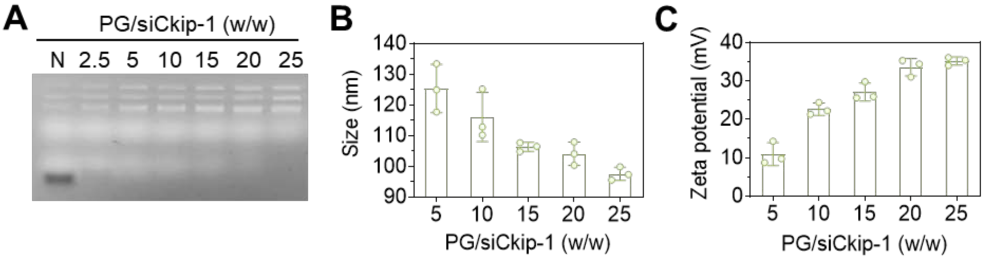

Figure S3. Agarose gel electrophoresis image (A), size (B) and zeta potential (C) of PsC NCs at various PG/siCkip-1 weight ratios (n = 3).

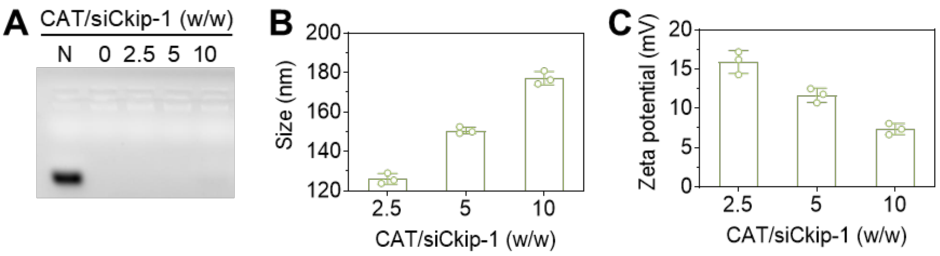

Figure S4. Agarose gel electrophoresis image (A), size (B), and zeta potential (C) of CPsC NCs at various CAT/siCkip-1 weight ratios (PG/siCkip-1 = 10, w/w, n = 3).

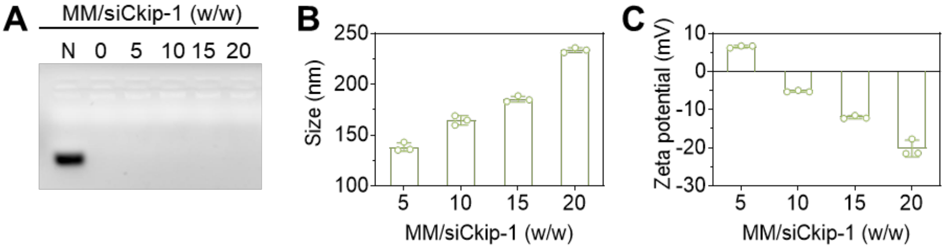

Figure S5. Agarose gel electrophoresis image (A), size (B), and zeta potential (C) of M@CPsC NCs at various MM/siCkip-1 weight ratios (CAT/siCkip-1 = 2.5, PG/siCkip-1 =10, w/w, *n* = 3).

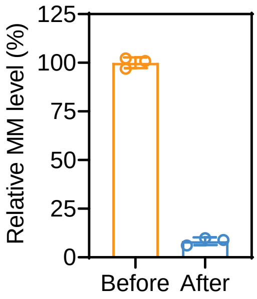

Figure S6. Relative MM levels of M@CPsC NCs (MM/CAT/PG/siCkip-1 = 10/2.5/10/1, w/w/w/w) composed of DiD-stained MM before or after centrifugation (10,000 g, 10 min) (*n* = 3).

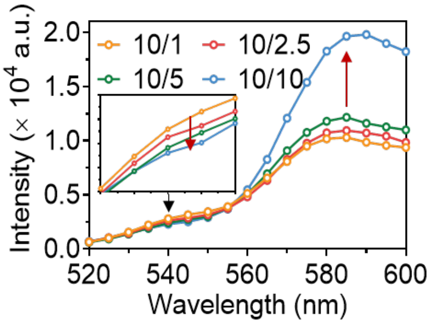

Figure S7. Fluorescence emission spectra (λ_{ex} = 480 nm) of ^{DS}M@CPsC NCs composed of DiO-stained MM and Cy3-labeled, DSPE-PEG$_{2k}$-conjugated sSDF-1α (Cy3-DS) at various membrane protein/sSDF-1α weight ratios. Black arrow points to the enlarged area.

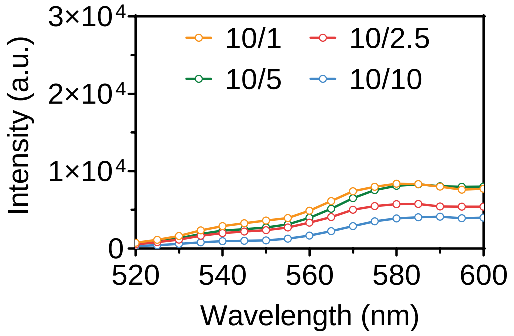

Figure S8. Fluorescence emission spectra (λ_{ex} = 480 nm) of the mixture of M@CPsC NCs (containing DiO-stained MM) and Cy3-labeled sSDF-1α (without DSPE as the membrane-anchoring domain) at various membrane protein/sSDF-1α weight ratios.

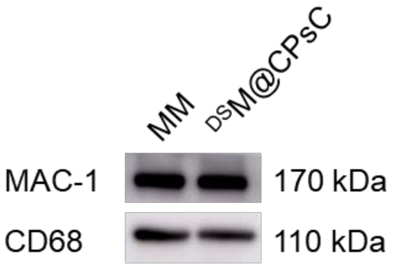

Figure S9. Western blot analysis of characteristic membrane markers of macrophages (MAC-1, CD68) in the isolated macrophage membrane (MM) and ^{DS}M@CPsC NCs.

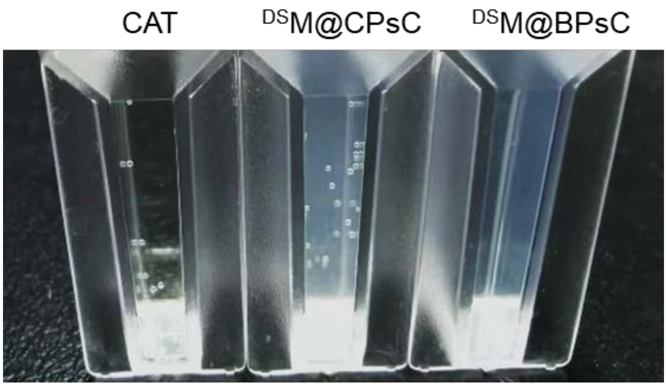

Figure S10. Photographs of CAT, ^{DS}M@CPsC NCs, and ^{DS}M@BPsC NCs (0.1 mg CAT or BSA/mL) after incubation with H_2O_2 (50 mM) for 1 h.

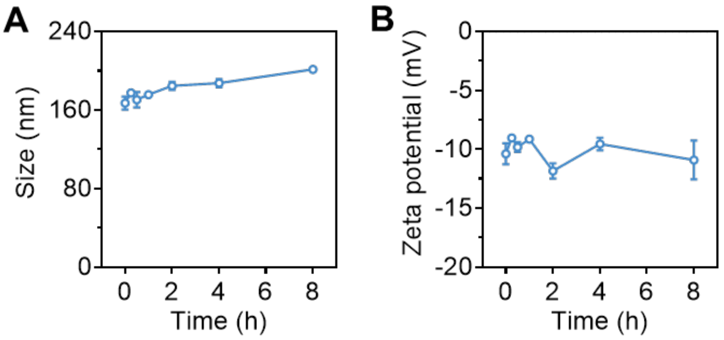

Figure S11. Size (a) and zeta potential (b) of ^{DS}M@BPsC NCs after treatment with H_2O_2 (100 μM) for various time (n = 3).

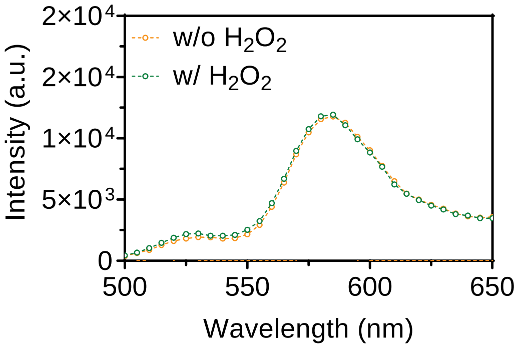

Figure S12. Fluorescence emission spectra (λ_{ex} = 494 nm) of FAM-siCkip-1-containing ^{DS}M@BPsC NCs with (w/) or without (w/o) H_2O_2 treatment (100 μM, 4 h). MM was pre-stained with DiI.

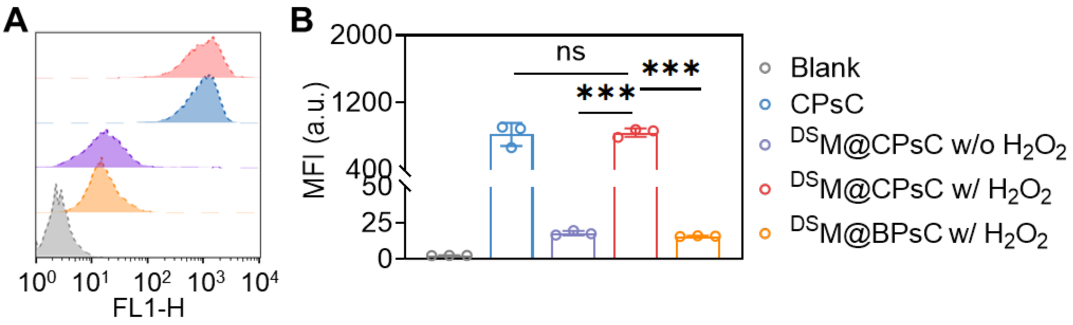

Figure S13. FCM histograms and mean fluorescence intensity of MSCs after 4-h incubation with various FAM-siCkip-1-containing NCs (n = 3).

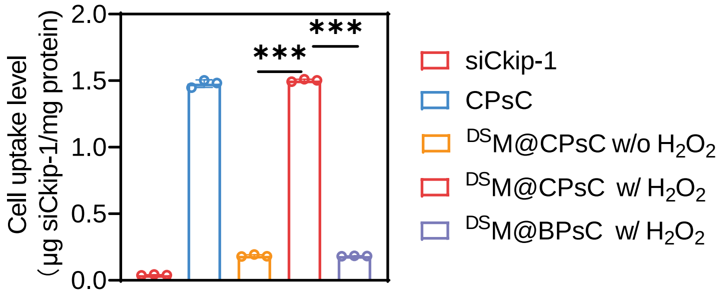

Figure S14. Uptake levels of various NCs containing FAM-siCkip-1 (1 μg siCkip-1/mL) in MSCs after incubation for 4 h (n = 3).

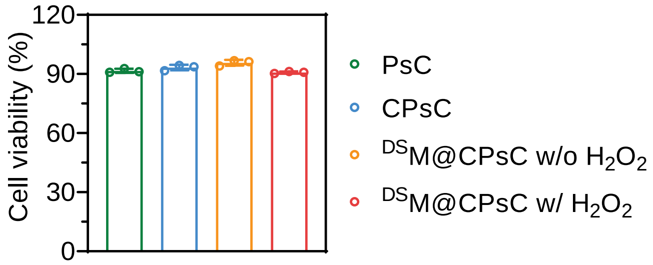

Figure S15. Cytotoxicity of PsC NCs, CPsC NCs, ^{DS}M@CPsC NCs, or H_2O_2 (100 μM, 4 h)-pretreated ^{DS}M@CPsC NCs in MSCs following 24-h incubation (n = 3).

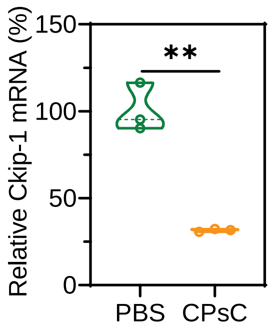

Figure S16. Ckip-1 mRNA levels in MSCs following CPsC NCs treatment (n = 3).

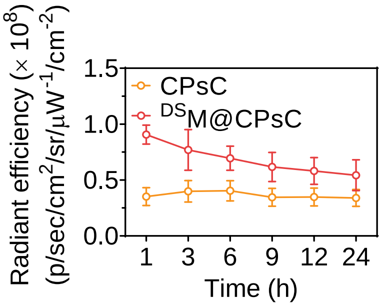

Figure S17. Calculated fluorescence intensities in the circled fracture regions in Figure 3b at various time points post *i.v.* injection of Cy5-siCkip-1-containing ^{DS}M@CPsC NCs or CPsC NCs (1 mg siCkip-1/kg, n = 3).

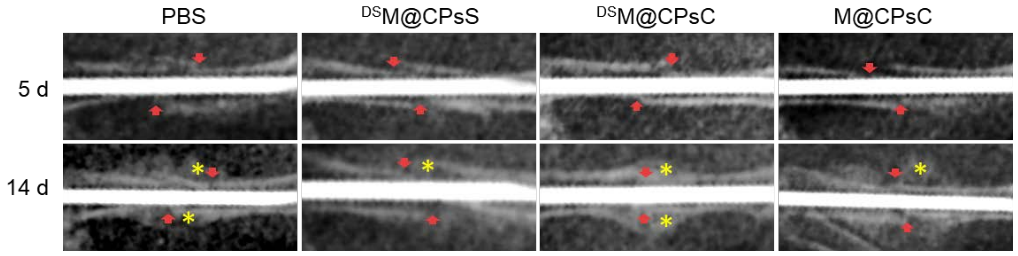

Figure S18. Representative X-ray radiographs of mice on day 5 and 14 post femur fracture. Stars represent callus, and red arrows indicate the fractured regions.

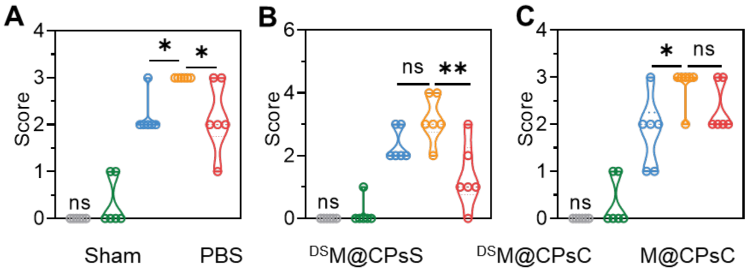

Figure S19. The scores of opacity (A), cortical remodeling and bridging (B), and periosteal and endosteal reaction (C) assessed from radiographic images on day 28 (n = 6).

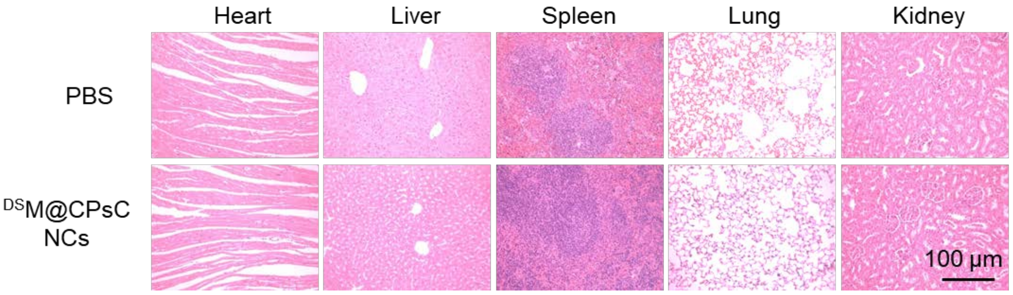

Figure S20. H&E staining of major organ sections (1 mg siCkip-1/kg, n = 3).

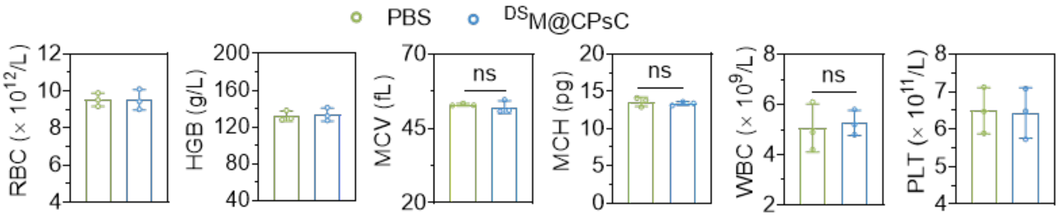

Figure S21. Hematological parameters of mice receiving three *i.v.* injections of PBS or ^{DS}M@CPsC NCs (1 mg siCkip-1/kg, n = 3).

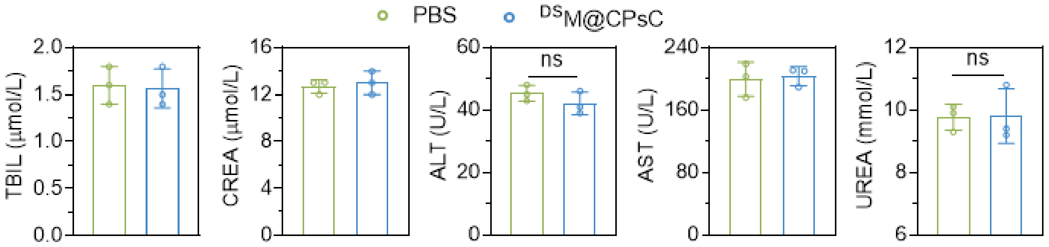

Figure S22. Serum biochemical levels of mice receiving three *i.v.* injections of PBS or ^{DS}M@CPsC NCs (1 mg siCkip-1/kg, n = 3).
